# Supplementary material for: Inter-site structural heterogeneity induction of single atom Fe catalysts for robust oxygen reduction
Source: Nat Commun. 2024 Mar 7;15:2062. doi: 10.1038/s41467-024-46389-3 (PMC10920901; doi:10.1038/s41467-024-46389-3)
Supplement: Supplementary file 1 — Supplementary Information [file 41467_2024_46389_MOESM1_ESM.pdf]

Supplementary Information

Inter-site structural heterogeneity induction of single atom Fe catalysts for robust oxygen reduction

Peng Zhang[1,6], Hsiao-Chien Chen[2,3,6], Houyu Zhu[4,6], Kuo Chen[1], Tuya Li[4], Yilin Zhao[1], Jiaye Li[1], Ruanbo Hu[5], Siying Huang[1], Wei Zhu[5], Yunqi Liu[1*] and Yuan Pan[1*]

[1]State Key Laboratory of Heavy Oil Processing, China University of Petroleum (East China), Qingdao 266580, China

[2]Center for Reliability Science and Technologies, Chang Gung University, Taoyuan 33302, Taiwan

[3]Kidney Research Center, Department of Nephrology, Linkou Chang Gung Memorial Hospital, Taoyuan, 333423, Taiwan

[4]School of Materials Science and Engineering, China University of Petroleum (East China), Qingdao 266580, China

[5]State Key Lab of Organic-Inorganic Composites, Beijing University of Chemical Technology, Beijing, 100029, China

[6]These authors contributed equally: Peng Zhang, Hsiao-Chien Chen, Houyu Zhu

*Corresponding author: liuyq@upc.edu.cn; panyuan@upc.edu.cn

This file includes:

Supplementary Figure 1-44

Supplementary Table 1-10

Supplementary references

Supplementary Figures

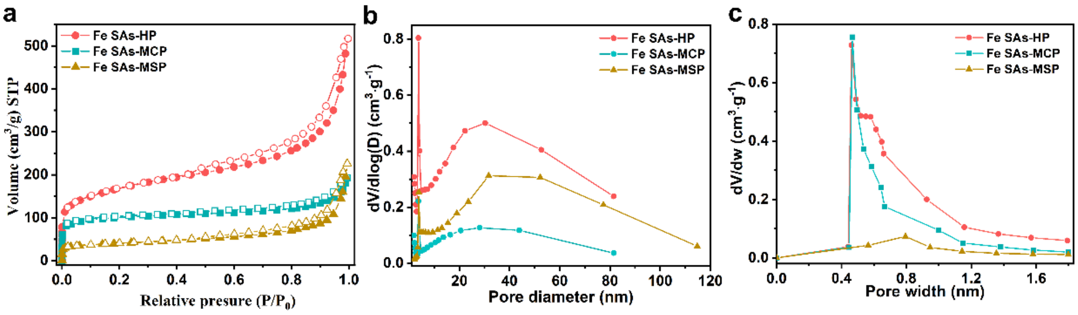

Supplementary Figure 1. N_2 adsorption-desorption isotherms and pore structure of as-prepared catalysts. (a) N_2 adsorption-desorption isotherms of as-prepared catalysts and (b)-(c) corresponding pore size distributions. The mesopore and micropore distributions were obtained from Barrett-Joyner-Halenda (BJH) and Horvath-Kawazoe methods, respectively.

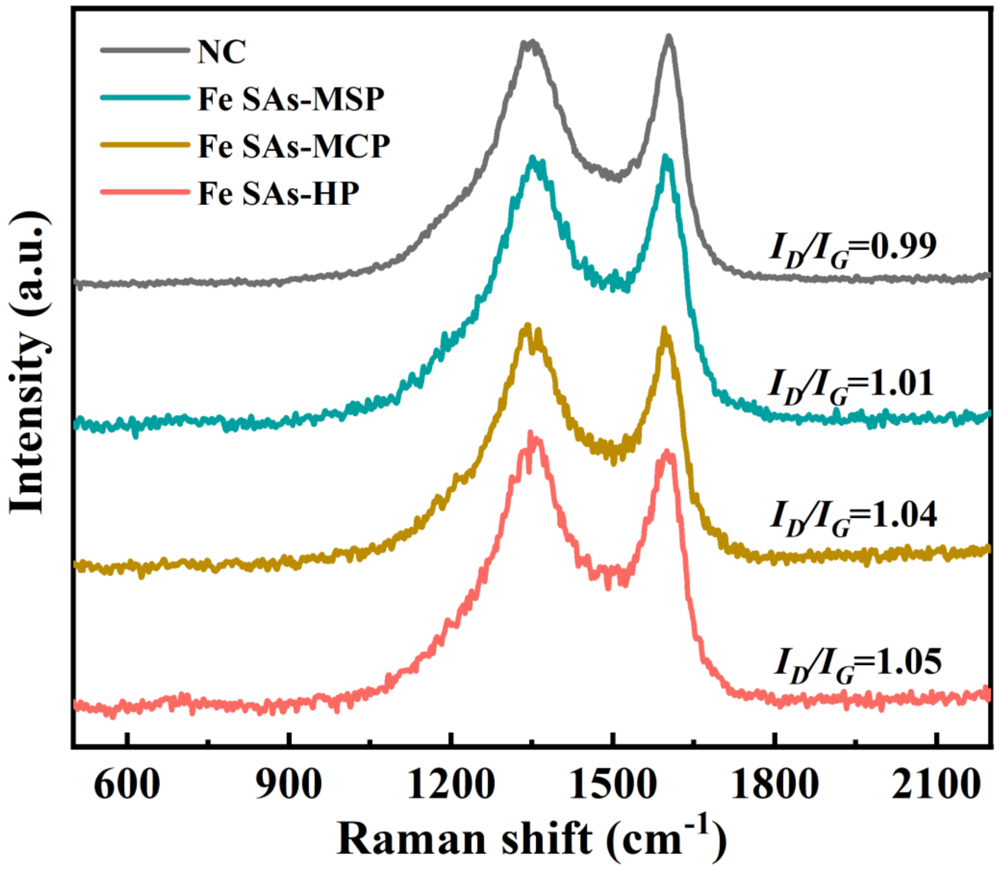

Supplementary Figure 2. Raman spectroscopy of prepared catalysts.

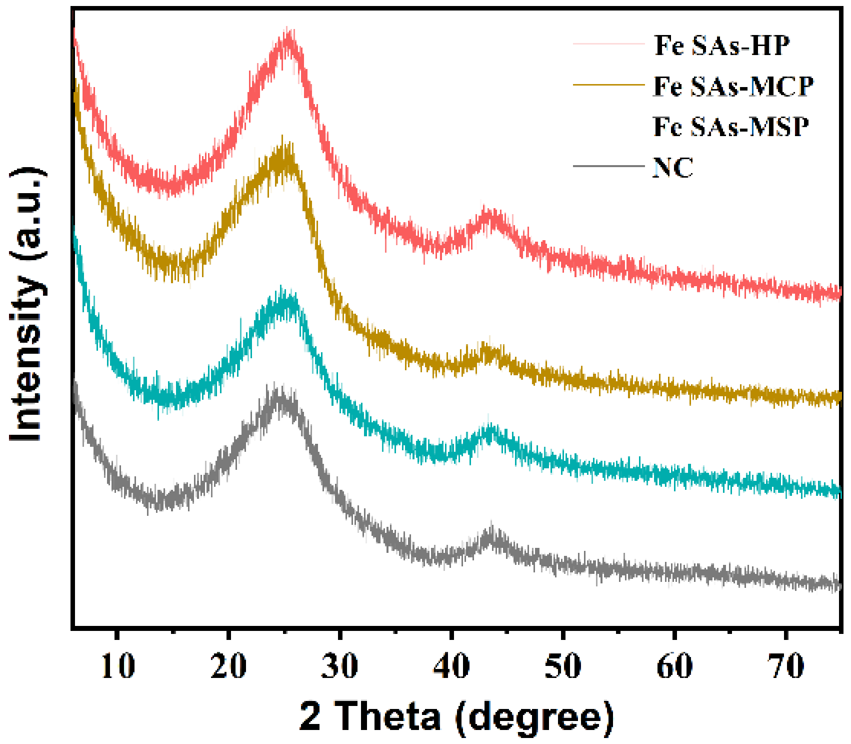

Supplementary Figure 3. XRD patterns of prepared catalysts. The catalysts were grinded into powders for XRD characterization.

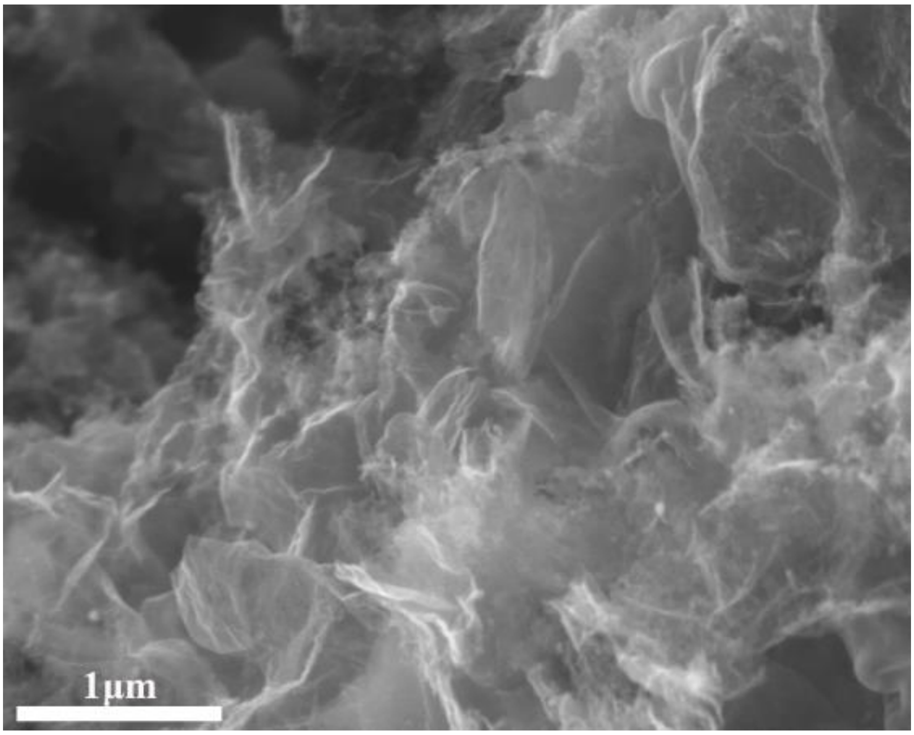

Supplementary Figure 4. SEM images of Fe SAs-HP. SEM images show the sheet-like microstructure of Fe SAs-HP.

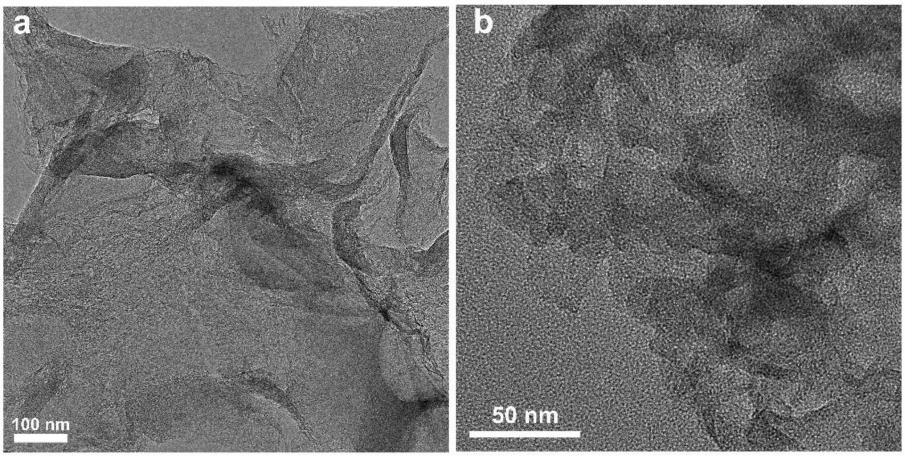

Supplementary Figure 5. TEM images. TEM images of (a) Fe SAs-MCP and (b) Fe SAs-MSP. Fe SAs-MCP exhibits sheet-like structure. Fe SAs-MSP exhibits sheet-like structure with dense pores as indicated by the bright dots.

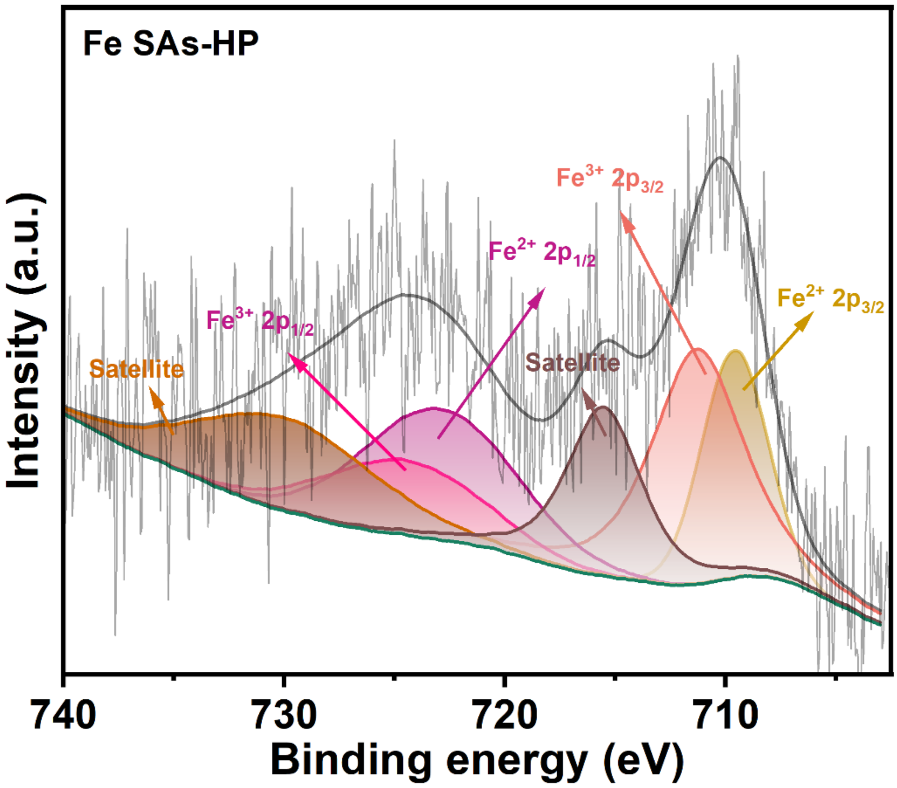

Supplementary Figure 6. XPS of Fe in Fe SAs-HP. Fe SAs-HP shows an oxidized valence state of Fe between +2 and +3.

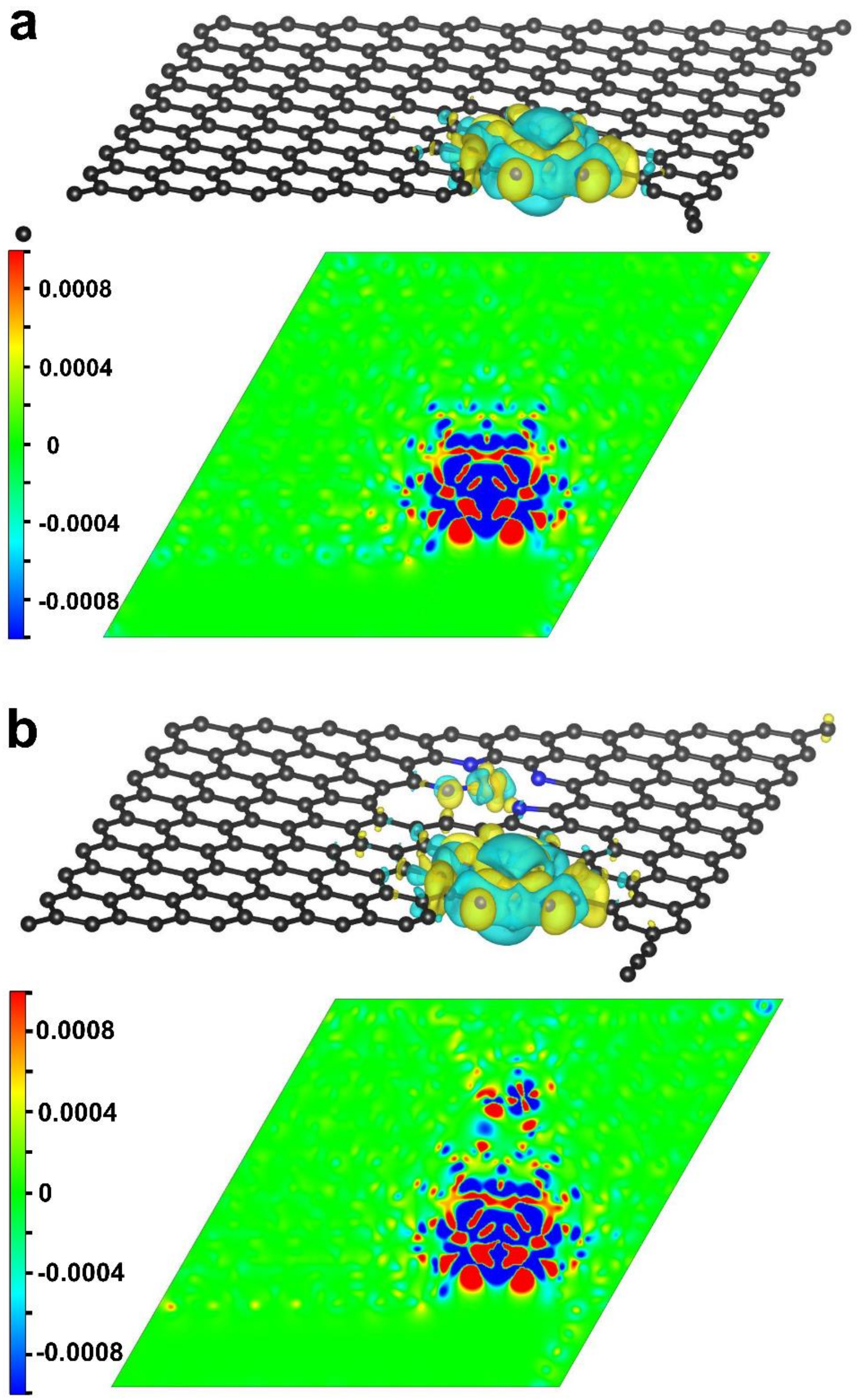

Supplementary Figure 7. Bader charge and differential charge densities. (a) Fe-N_4 at mesoporous edge. (b) incorporating microporous Fe-N_4 in the vicinity of mesoporous FeN_4. Fe atoms at mesoporous edges in structure a will lose 1.24 e^-. In the case of microporous Fe-N_4 sites, mesoporous Fe atoms will lose 1.25 e^- and microporous Fe-N_4 will lose 1.2 e^-.

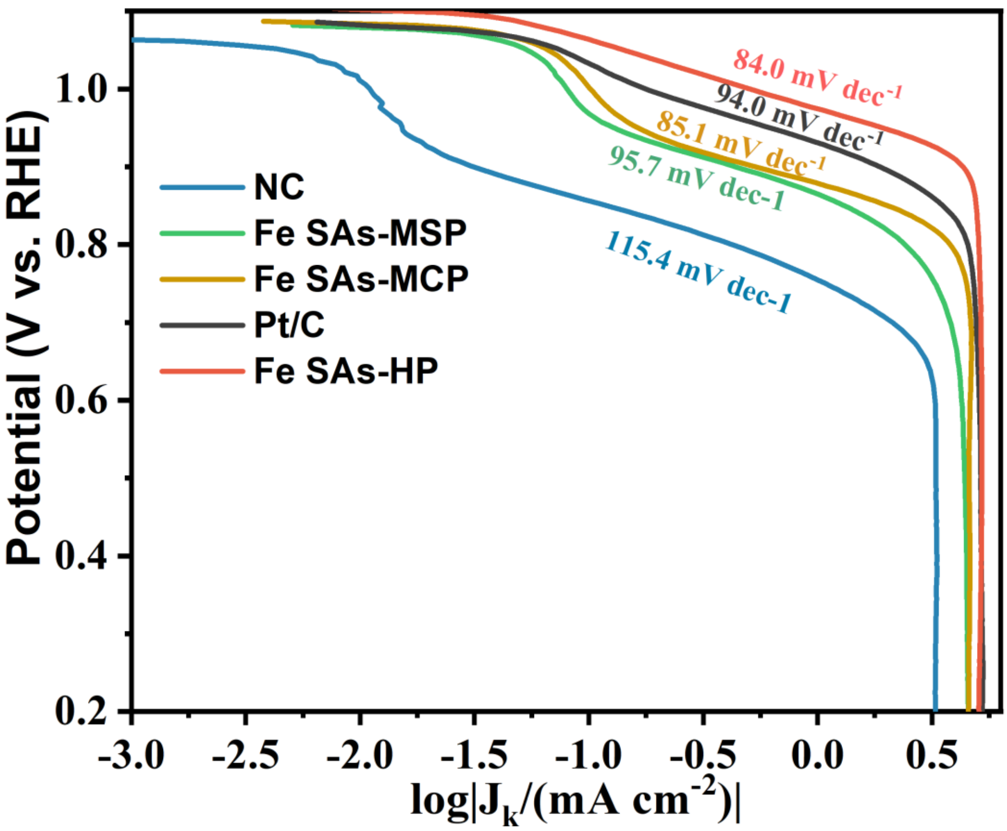

Supplementary Figure 8. Tafel slopes of as-prepared catalysts in alkaline medium. Fe SAs-HP exhibits a relative low Tafel slope of 84.0 mV dec^{-1}, indicating a faster kinetic.

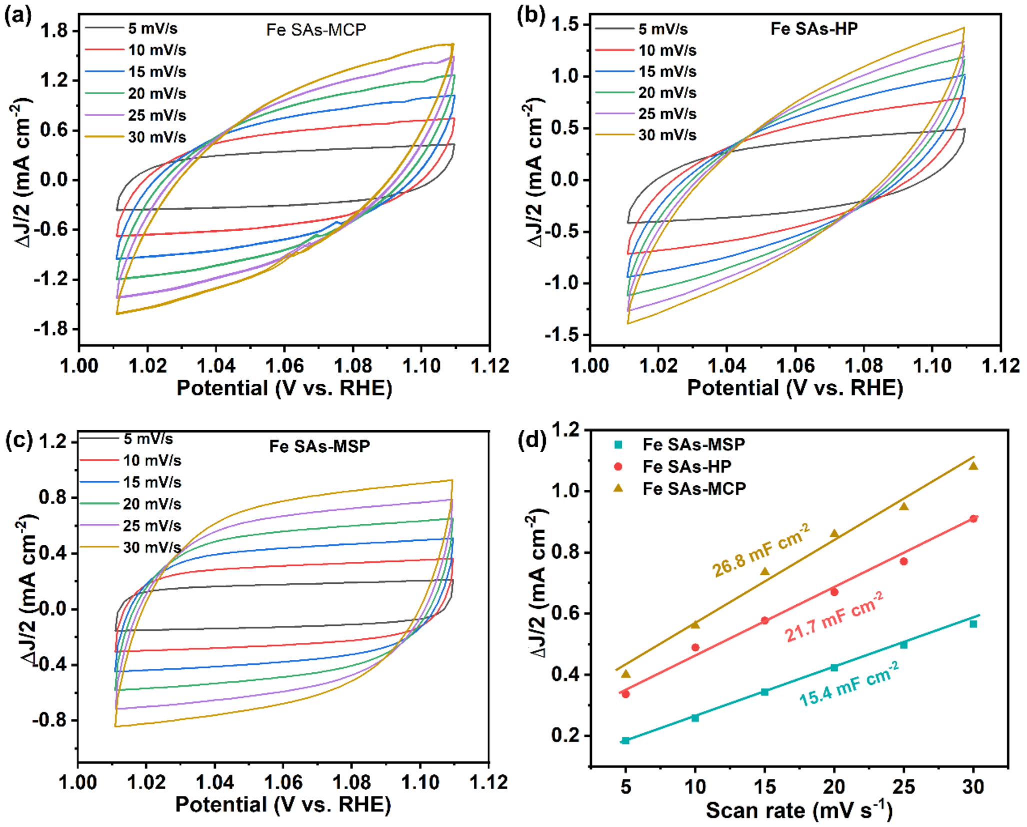

Supplementary Figure 9. CV curves at different scan rates and double-layer capacitance (C_{dl}) of prepared catalysts. (a) CV for Fe SAs-MCP. (b) CV for Fe SAs-HP. (c) CV for Fe SAs-MSP. (d)double-layer capacitance. The C_{dl} is in proportion to electrochemical active surface area (ECSA). Fe SAs-MCP has the highest C_{dl} and ECSA.

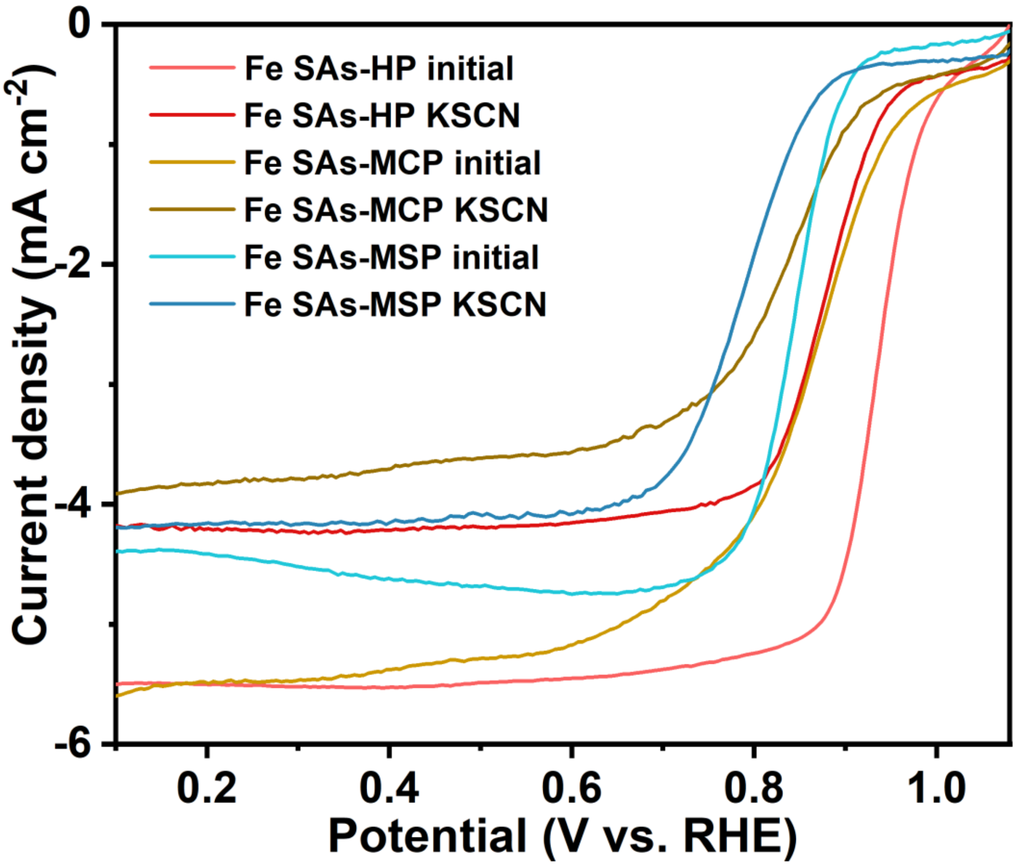

Supplementary Figure 10. ORR polarization curves of as-prepared catalysts before and after adding KSCN solutions for 30 min. The scan rate is 5 mV/s.

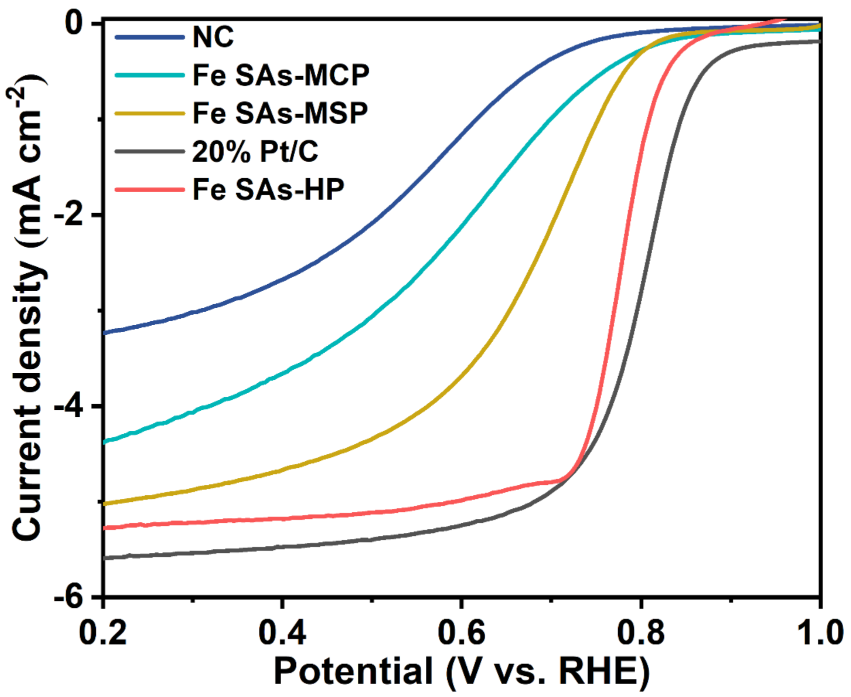

Supplementary Figure 11. LSV of as-prepared catalysts measured in 0.1 M $HClO_4$. The catalysts loadings are kept at 1.0 mg cm^{-2}.

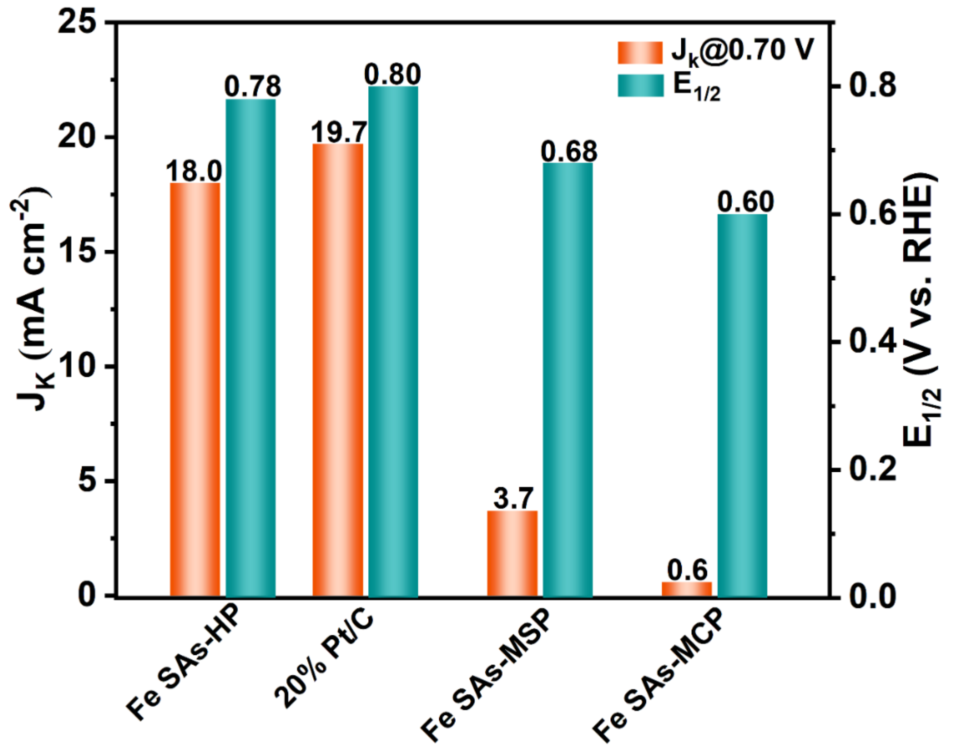

Supplementary Figure 12. Comparison of kinetic current density and $E_{1/2}$ of as-prepared catalysts measured in 0.1 M $HClO_4$.

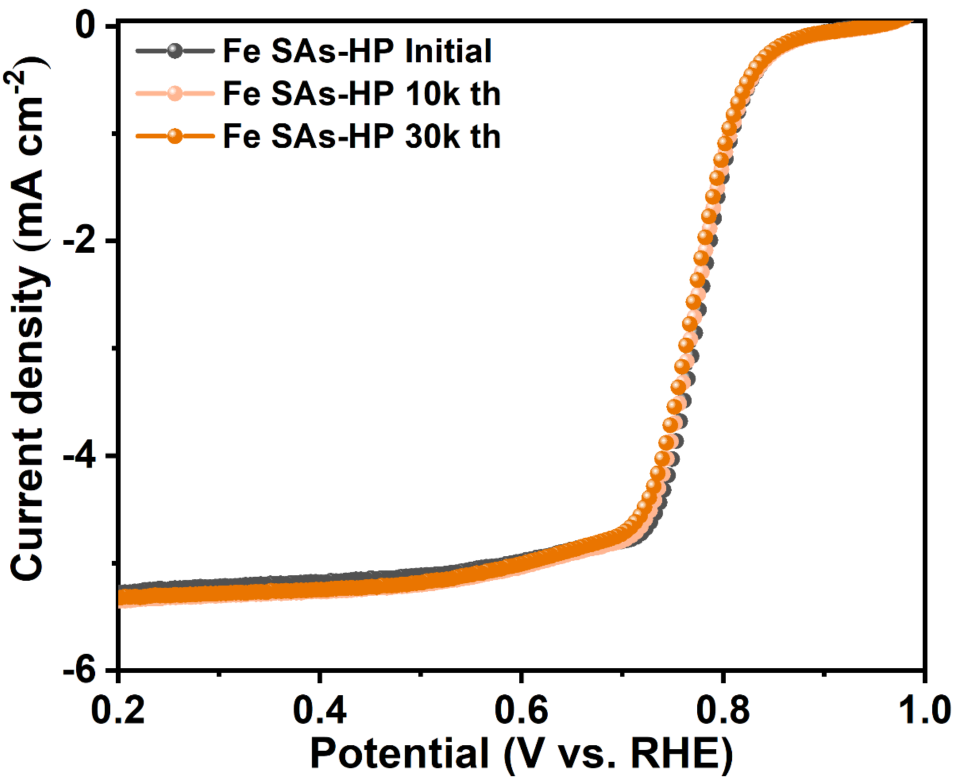

Supplementary Figure 13. LSV of Fe SAs-HP after ADT test in 0.1 M $HClO_4$.

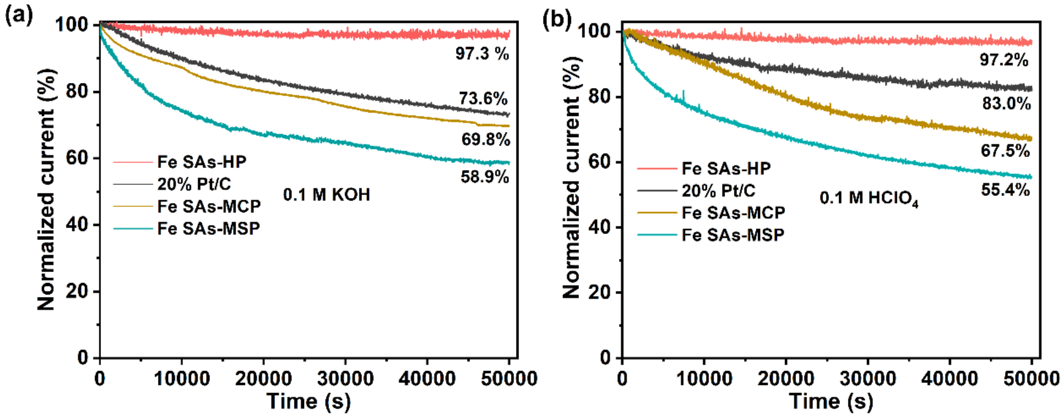

Supplementary Figure 14. Long-term durability tests. (a) and (b) i-t chronoamperometric tests of prepared catalysts at 0.75 V under 0.1 M KOH and 0.1 M $HClO_4$, respectively. Fe SAs-HP shows the highest current retention in both alkaline and acidic electrolytes after 50000s tests.

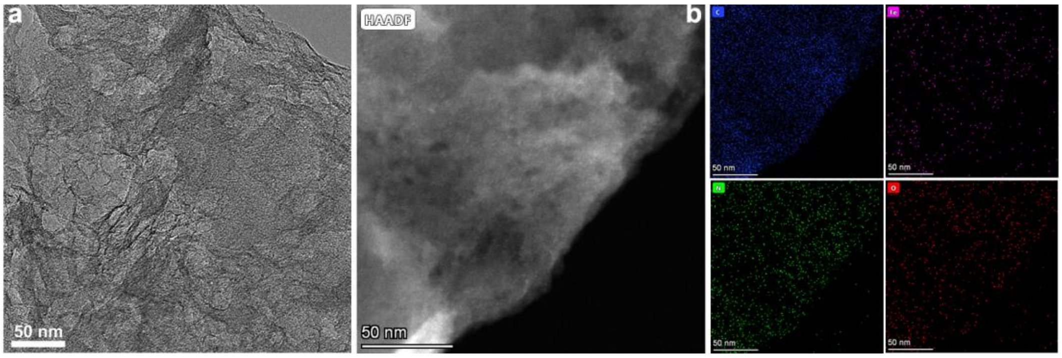

Supplementary Figure 15. TEM images and corresponding EDS element mapping of Fe SAs-HP after ADT test. (a) TEM image. (b) HAADF-STEM and corresponding EDS element mapping of C, Fe, N and O, respectively. No obvious agglomeration of metal particles can be observed.

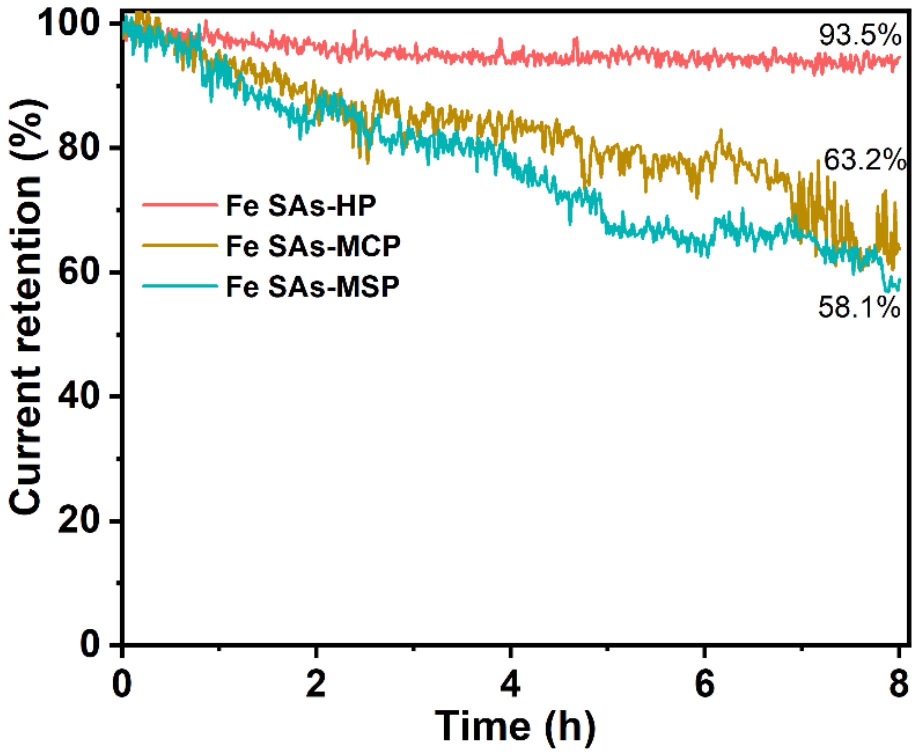

Supplementary Figure 16. Harsh i-t chronoamperometric tests of prepared catalysts at 0.2 V under 20 mL of O_2 saturated 0.1 M KOH. Fe SAs-HP exhibits current retention of 93.5 %. The violent fluctuate of tested curves were ascribed to the limited volume of electrolytes.

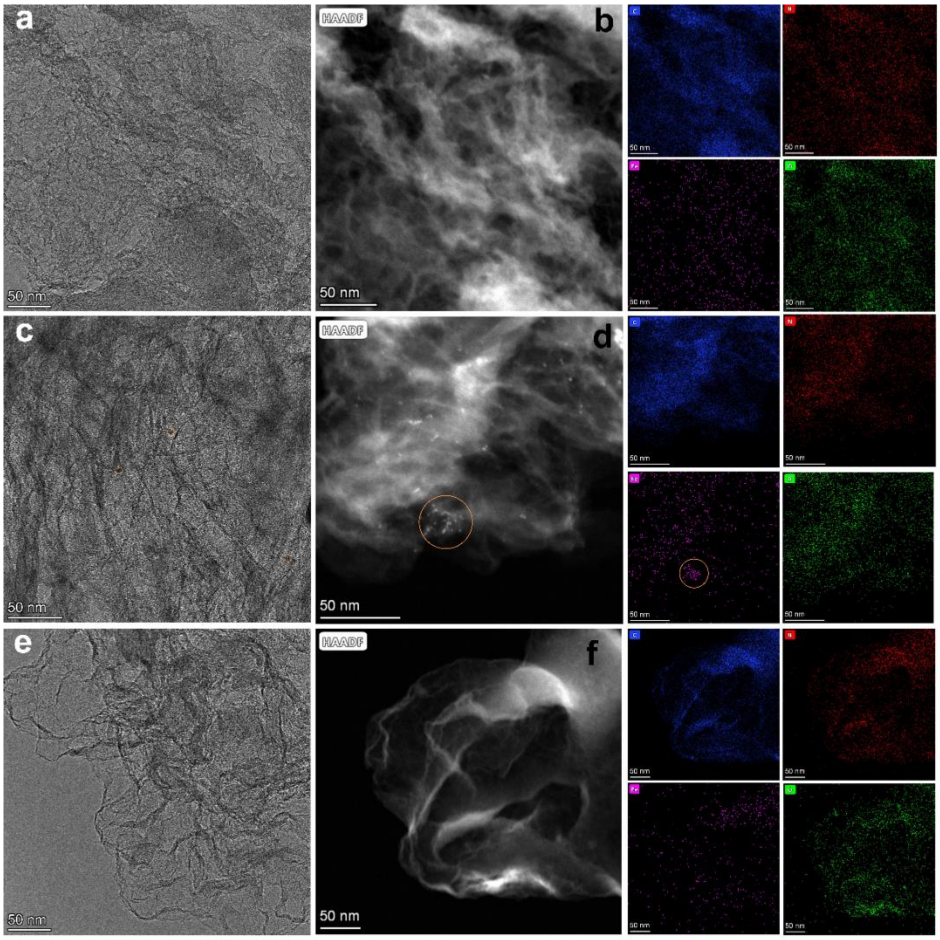

Supplementary Figure 17. High resolution TEM, HAADF STEM and corresponding element mapping after harsh durability tests. (a) and (b) Fe SAs-HP; (c) and (d) Fe SAs-MCP; (e) and (f) Fe SAs-MSP. Fe SAs-HP and Fe SAs-MSP show no obvious aggregation of metal particles. However, tiny metal clusters can be observed for Fe SAs-MCP after harsh durability tests.

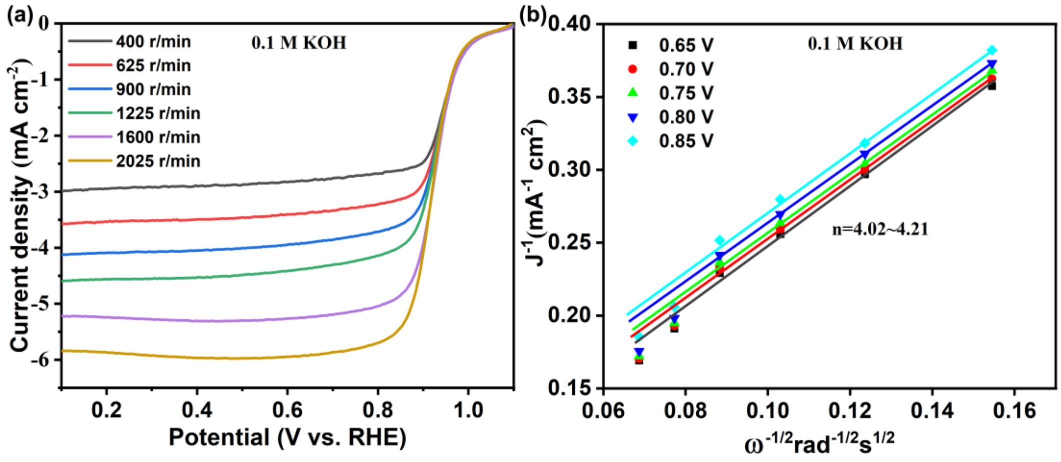

Supplementary Figure 18. LSV curves at different rotations and charge transfer number in 0.1 M KOH. (a) ORR polarization curves of Fe SAs-HP at different rotations under 0.1 M KOH and (b) corresponding charge transfer number. Fe SAs-HP exhibits 4-e^- ORR process under alkaline electrolytes.

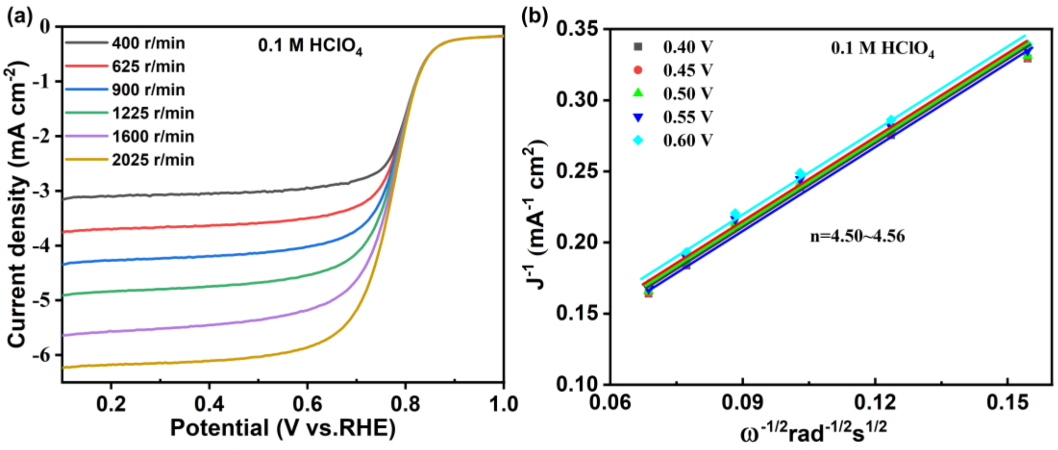

Supplementary Figure 19. LSV curves at different rotations and charge transfer number in 0.1 M $HClO_4$. (a) ORR polarization curves of Fe SAs-HP at different rotations under 0.1 M $HClO_4$ and (b) corresponding charge transfer number. Fe SAs-HP exhibits an approximate 4-e^- ORR process under acidic electrolytes.

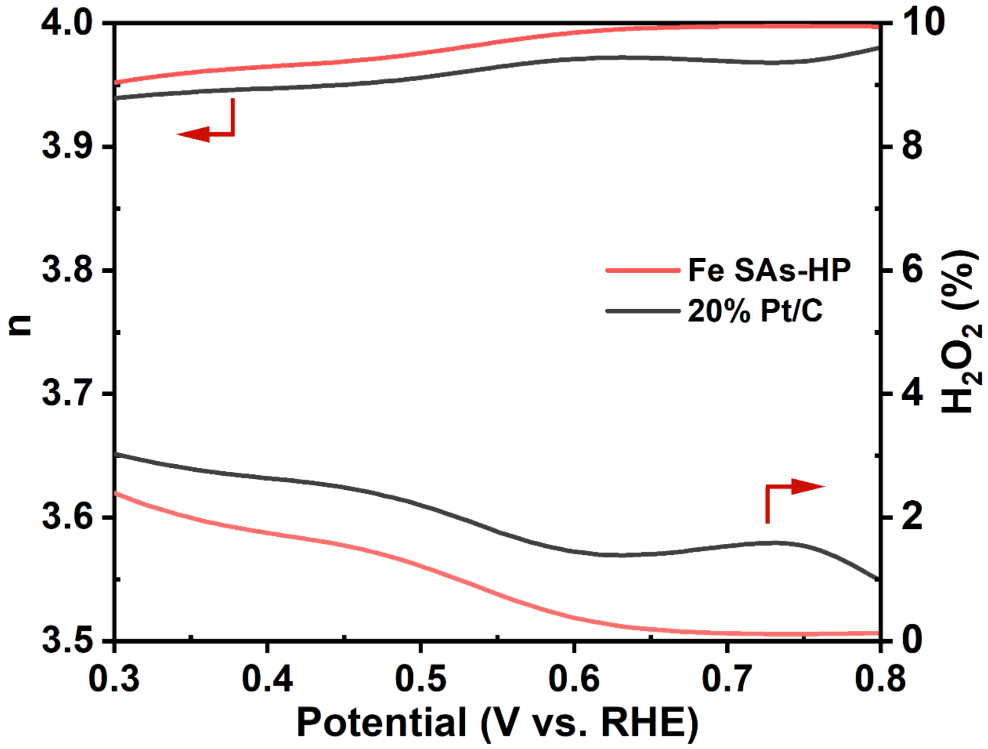

Supplementary Figure 20. Charge transfer number and H_2O_2 yield of Fe SAs-HP and 20% Pt/C by RRDE test. Fe SAs-HP shows 4-e^- ORR process with low H_2O_2 yield.

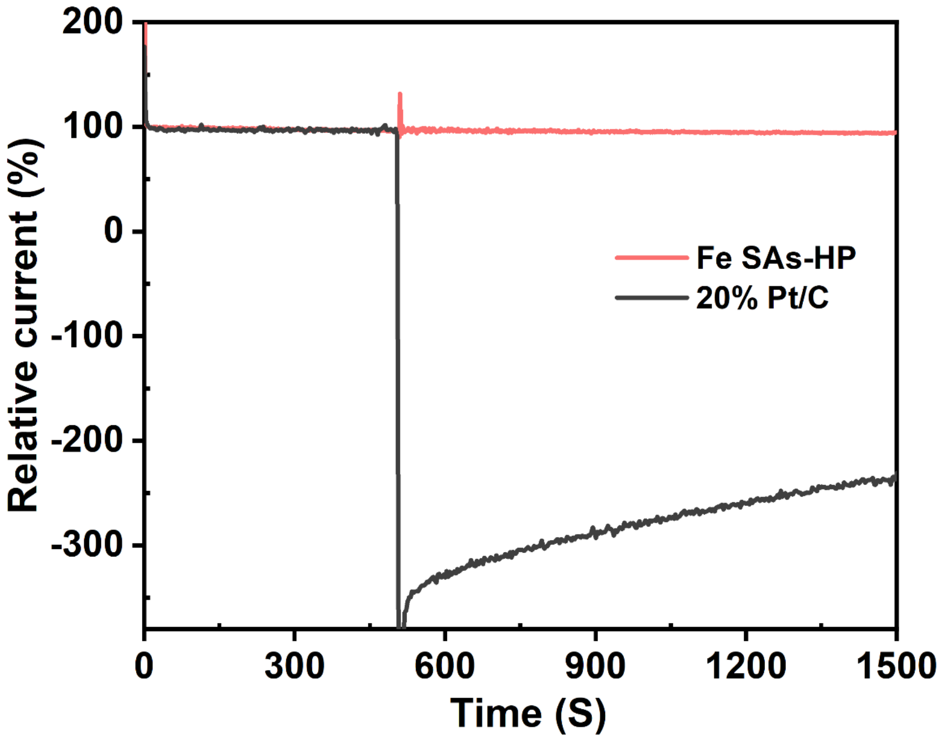

Supplementary Figure 21. Methanol tolerance test of Fe SAs-HP and 20% Pt/C. The methanol was injected at 500 s.

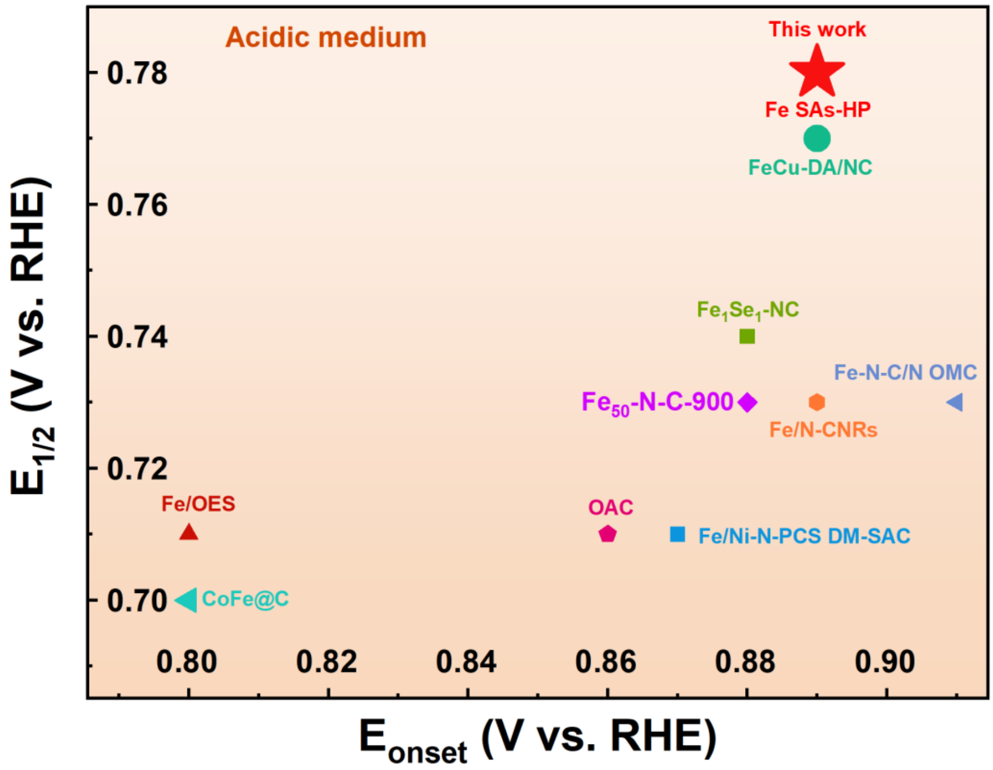

Supplementary Figure 22. Comparison of acidic ORR performance for Fe SAs-HP with reported catalysts. The reported catalysts are summarized in supplementary table 5.

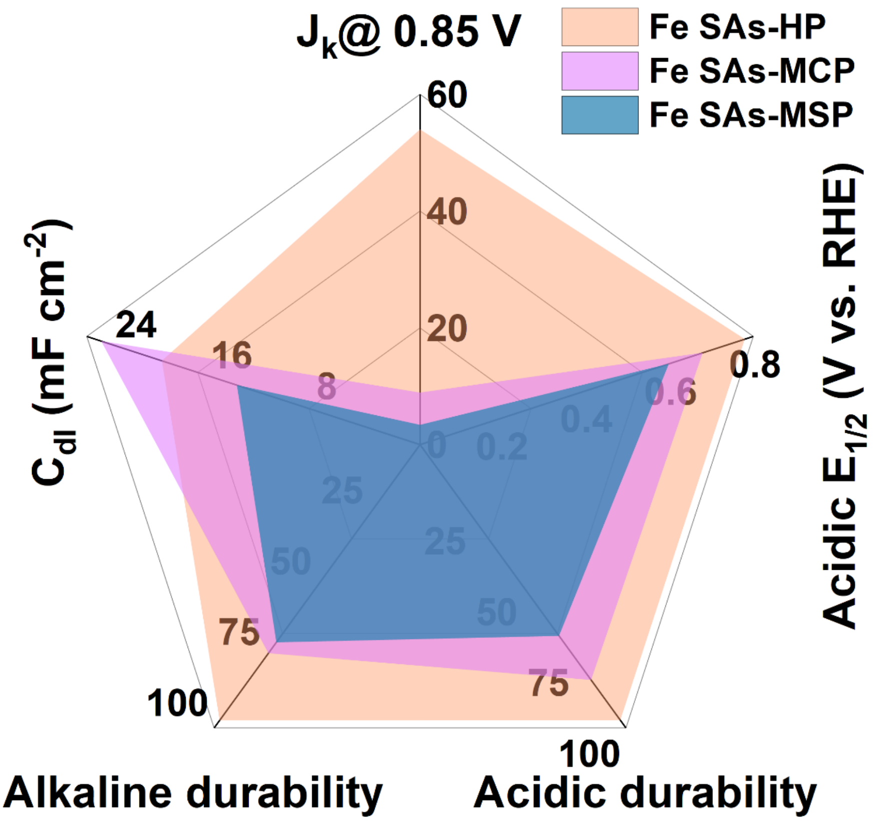

Supplementary Figure 23. Comprehensive performance of as-prepared catalysts with different pore structure.

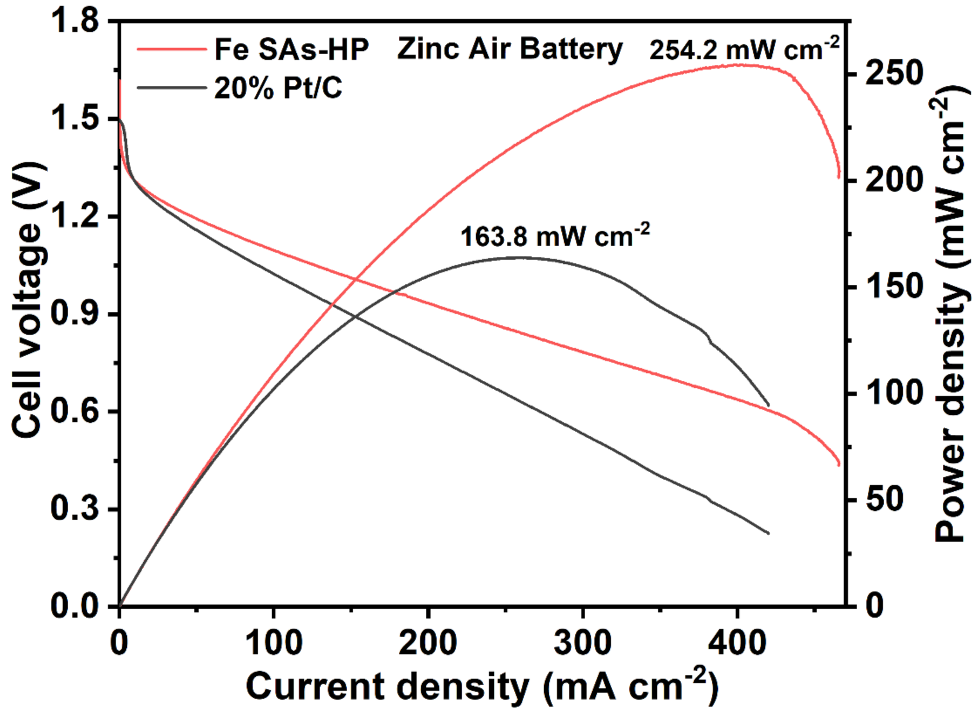

Supplementary Figure 24. Polarization curves and corresponding power density of assembled zinc air batteries with Fe SAs-HP and commercial Pt/C as air cathodes. Fe SAs-HP exhibits peak power density of 254.2 mW cm^{-2}.

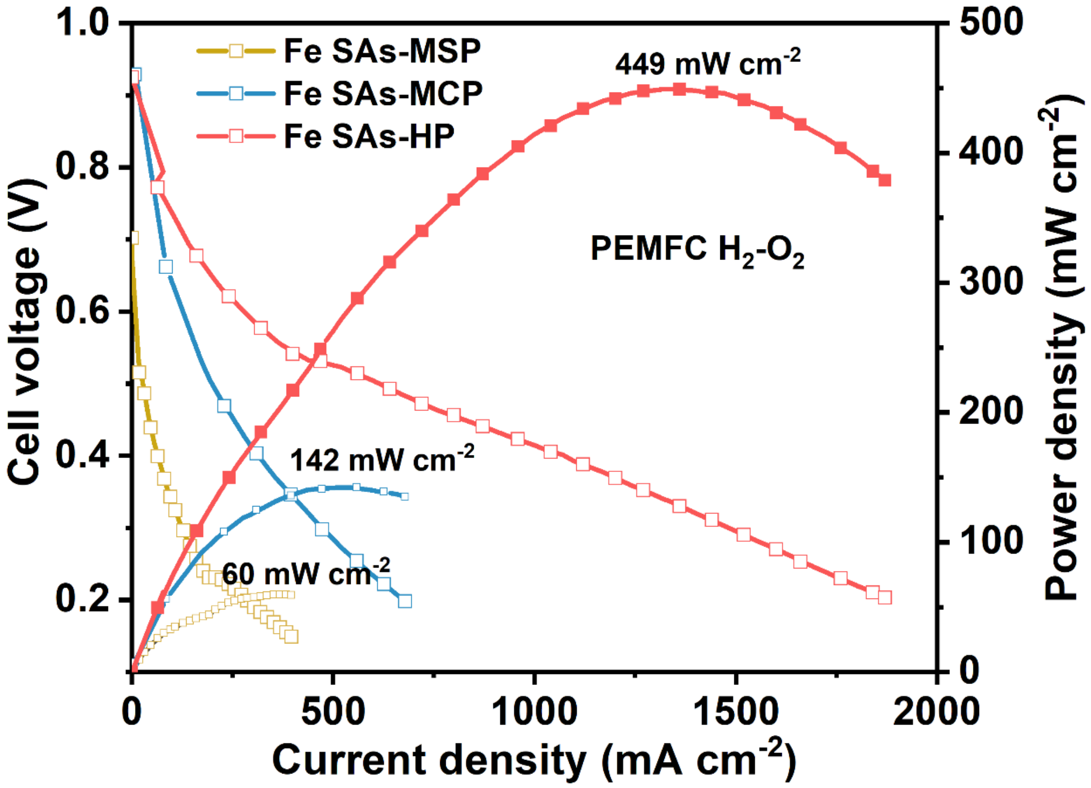

Supplementary Figure 25. Polarization curves and corresponding power density of assembled H_2-O_2 PEMFC of prepared catalysts. Fe SAs-HP exhibits peak power density of 449 mW cm^{-2}, followed by Fe SAs-MCP and Fe SAs-MSP.

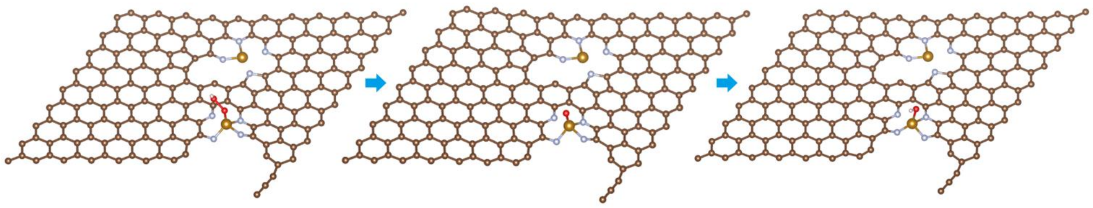

Supplementary Figure 26. Simulated ORR reaction pathway of Fe SAs-HP at mesoporous Fe-N_4 sites (Fe SAs-HP@MSP).

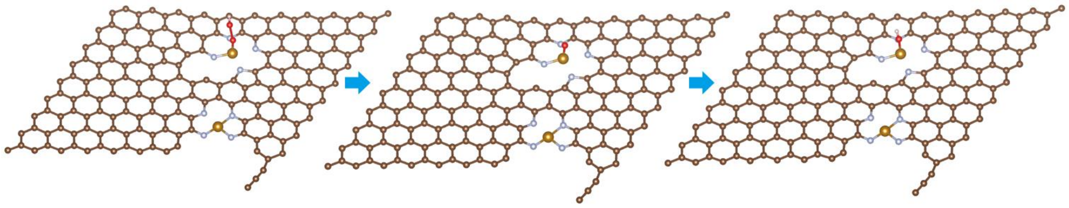

Supplementary Figure 27. Simulated ORR reaction pathway of Fe SAs-HP at microporous Fe-N_4 sites (Fe SAs-HP@MCP)

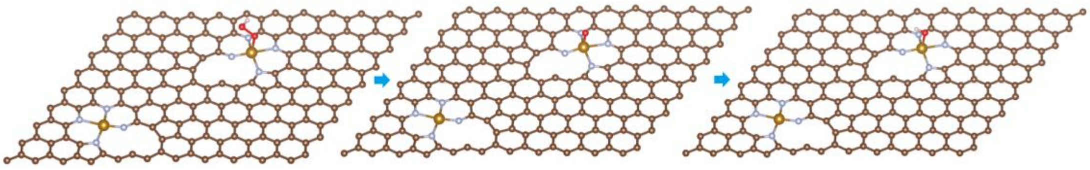

Supplementary Figure 28. Simulated ORR reaction pathway of Fe SAs-MCP on microporous Fe-N_4 sites (Fe SAs-MCP)

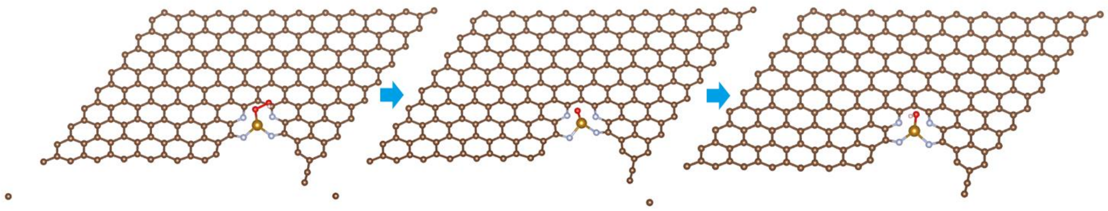

Supplementary Figure 29. Simulated ORR reaction pathway of Fe SAs-MSP on mesoporous Fe-N_4 sites (Fe SAs-MSP)

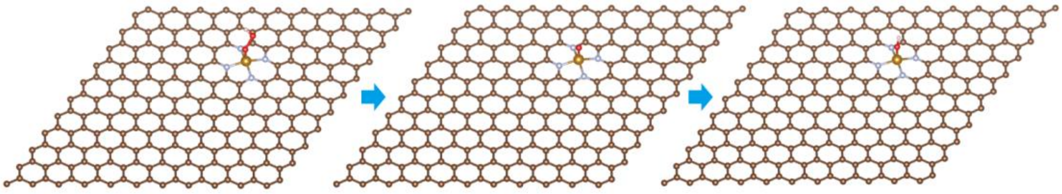

Supplementary Figure 30. Simulated ORR reaction pathway of conventional in-plane type Fe-N_4 sites (FeN_4-in plane)

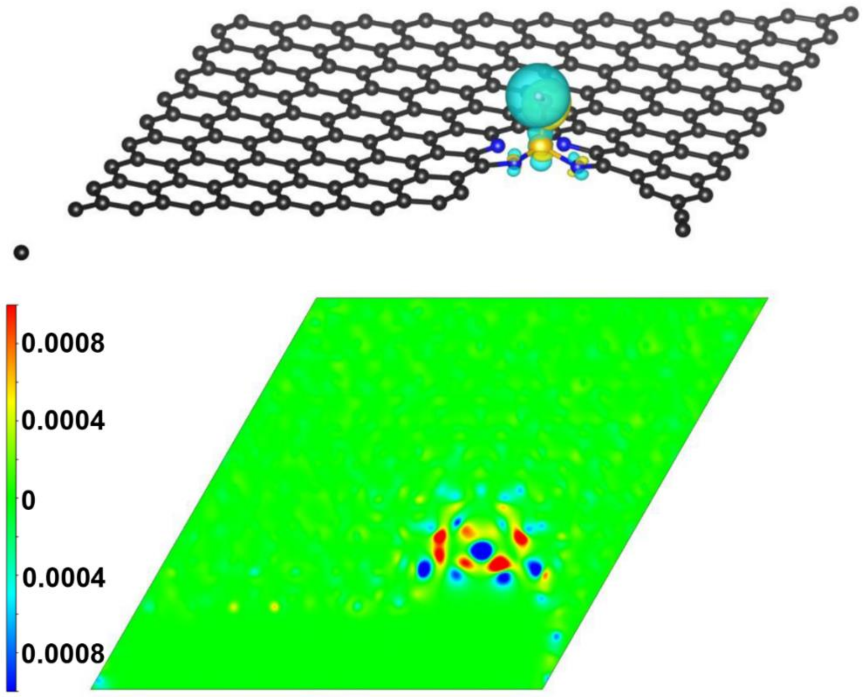

Supplementary Figure 31. Differential charge densities of single mesoporous $Fe\text{-}N_4$ sites after adsorption of *OH. Central Fe atoms will lose 1.379 e^-, while *OH will gain 0.497 e^-.

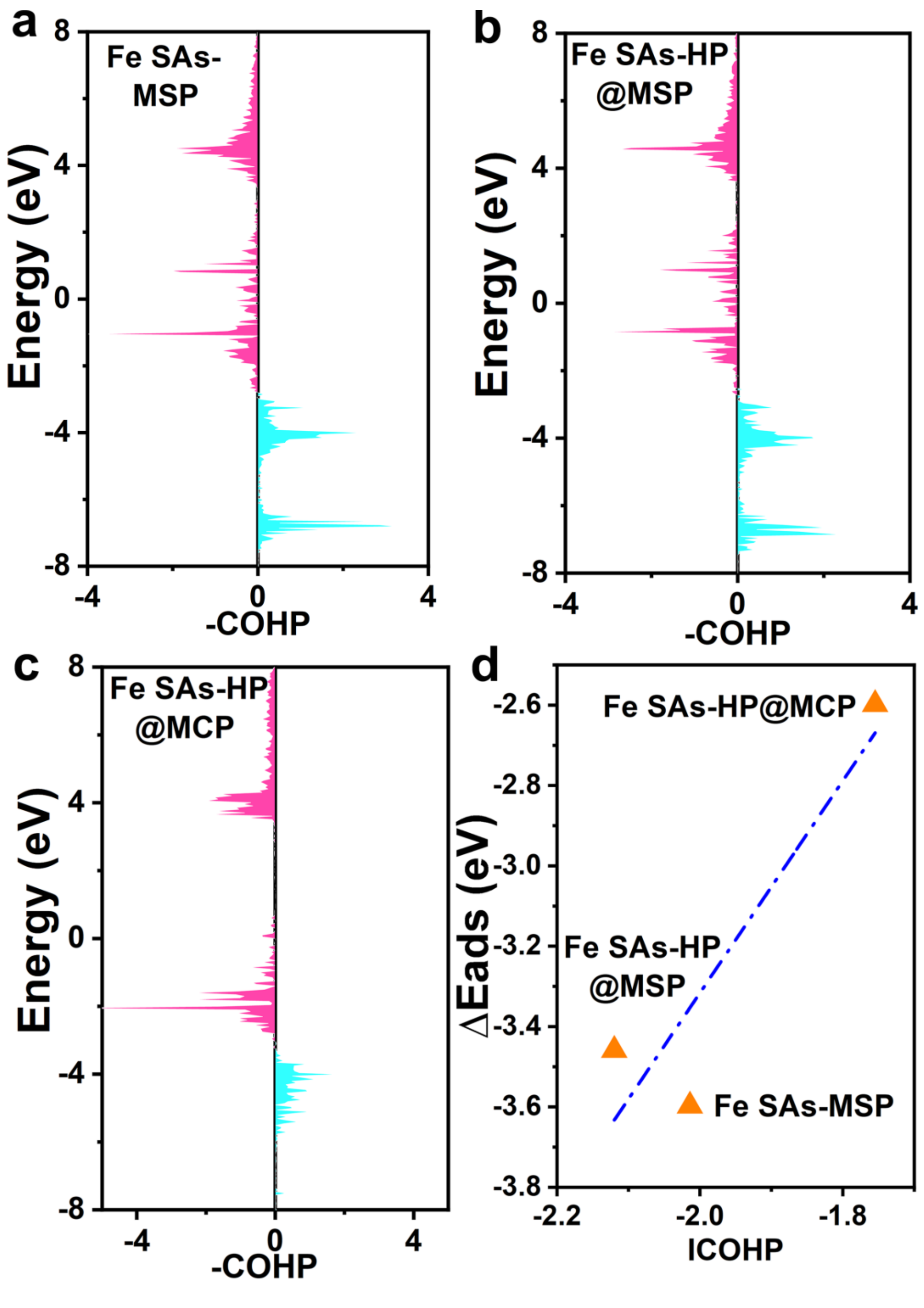

Supplementary Figure 32. The projected crystal orbital Hamilton population (COHP) and integrated COHP (ICOHP) of as-prepared catalysts. (a) Fe SAs-MSP. (b) Fe SAs-HP@MSP. (c) Fe SAs-HP@MCP. (d) ICOHP. Fe SAs-HP@MSP has the optimized adsorption for *OH.

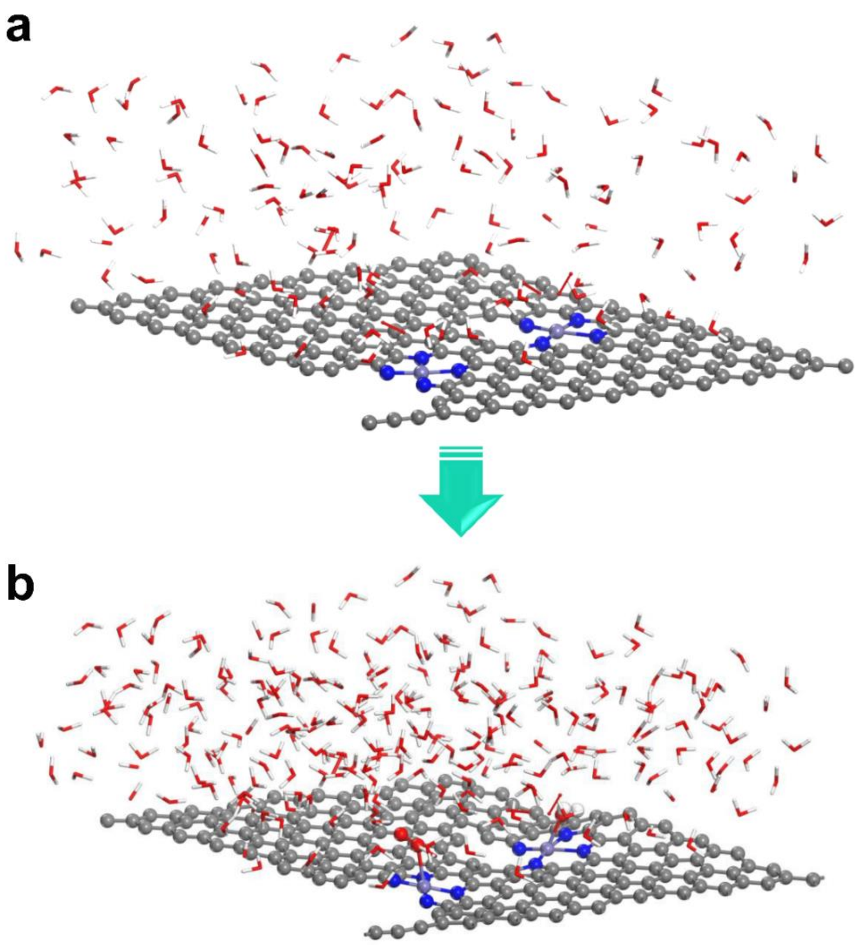

Supplementary Figure 33. AIMD simulated interfacial structure of Fe SAs-HP. (a) initial state and (b) final state of dynamic adsorption. The O_2 molecules are preferentially adsorbed on mesoporous sites. And H_2O molecules are adsorbed on microporous sites.

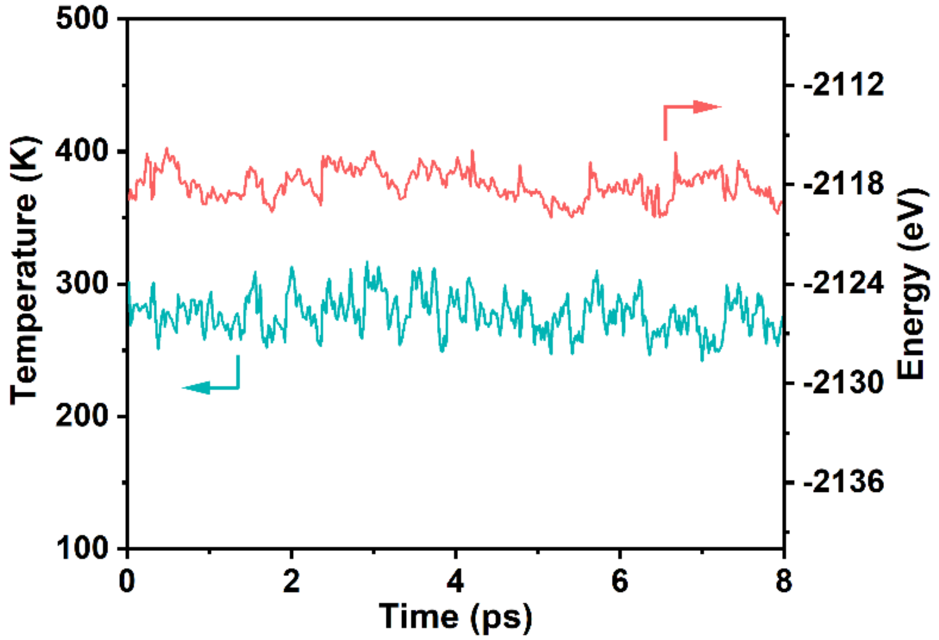

Supplementary Figure 34. Energy and temperature fluctuation during simulations. Energy and temperature fluctuated within a certain range.

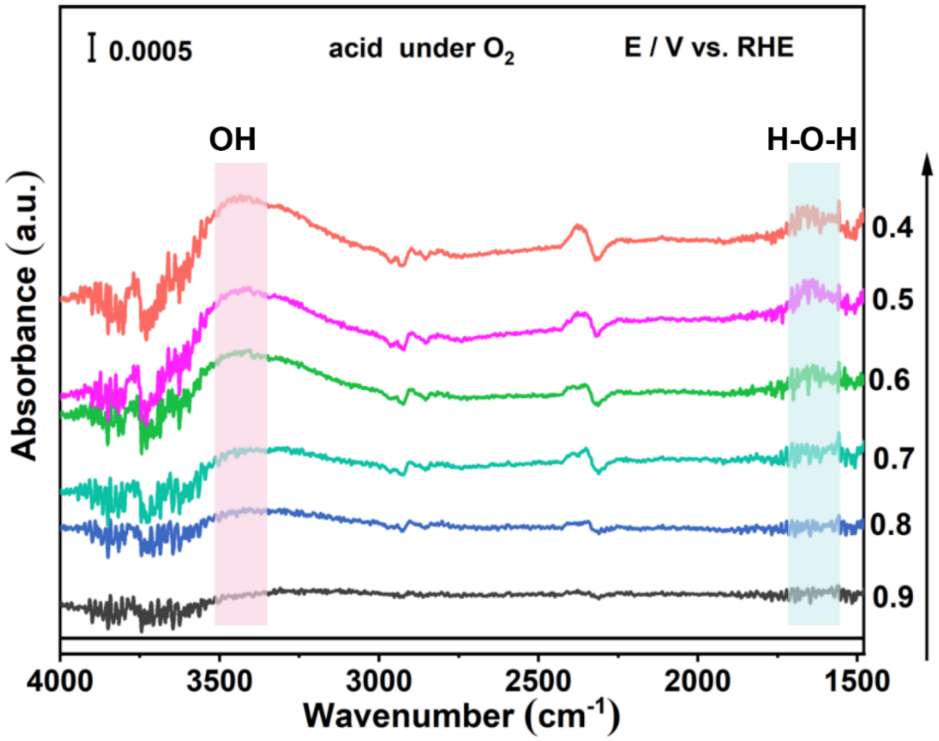

Supplementary Figure 35. *In situ* ATR-SEIRAS of Fe SAs-HP under acidic media. The adsorption of OH and H-O-H increased with applied overpotentials.

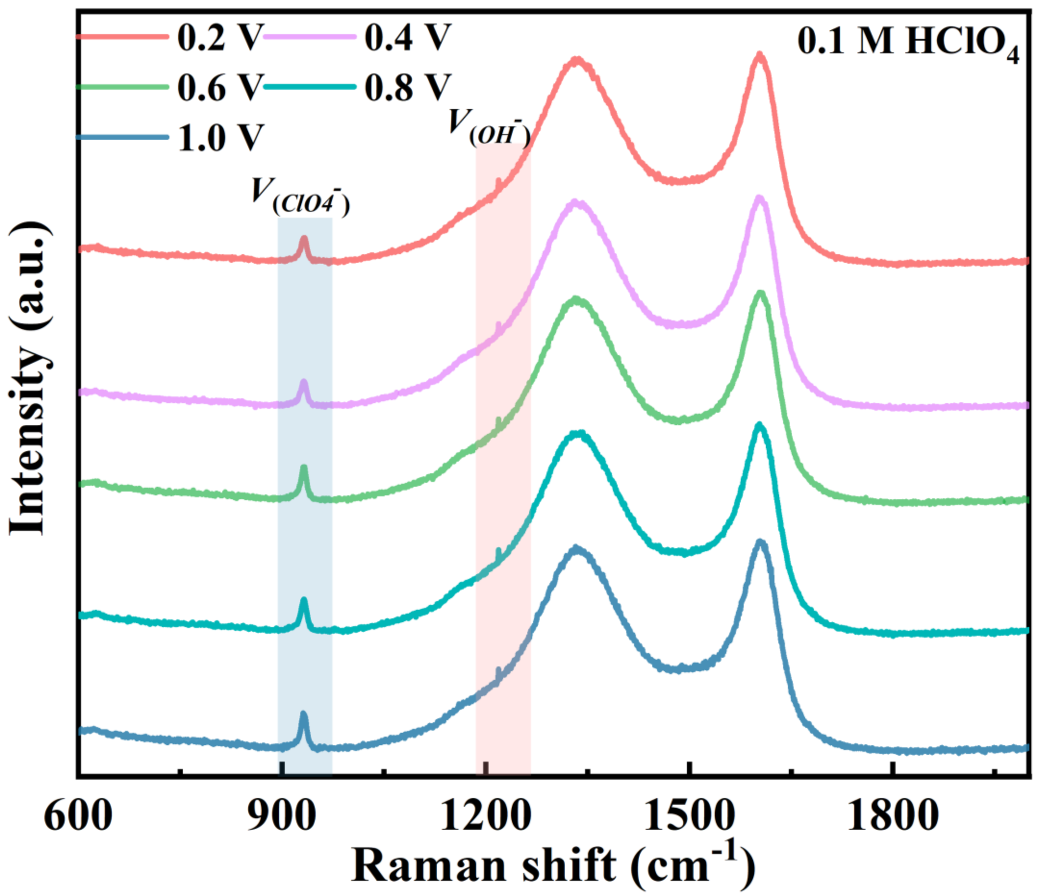

Supplementary Figure 36. *In situ* Raman spectra of Fe SAs-HP tested in 0.1 M $HClO_4$. The vibration of ClO_4^- and OH^- can be observed.

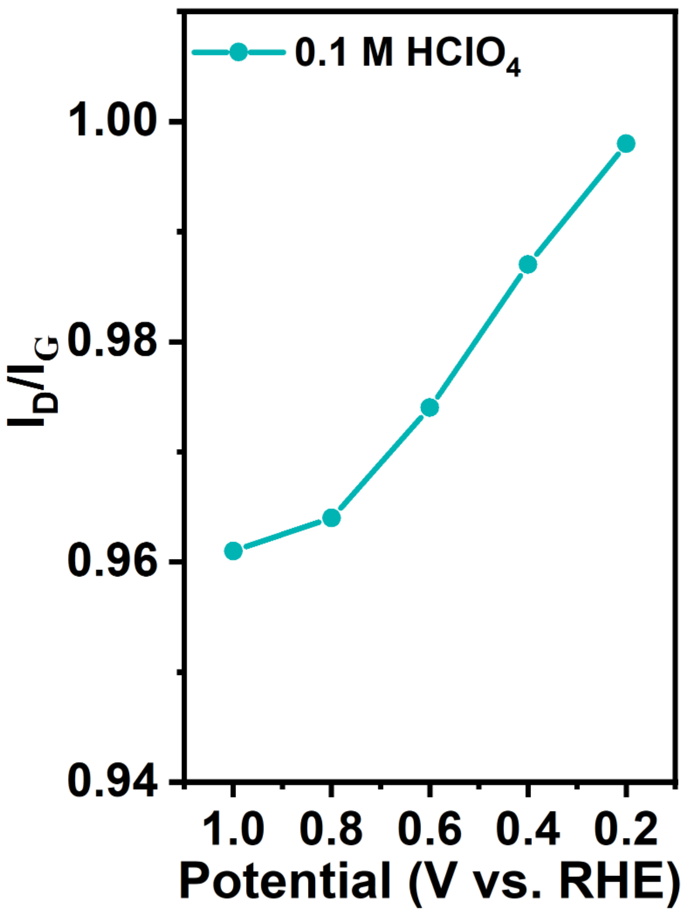

Supplementary Figure 37. I_D/I_G values with applied overpotentials under acidic working conditions. I_D/I_G shifted to higher values from 0.96 to 1.00 with applied overpotentials.

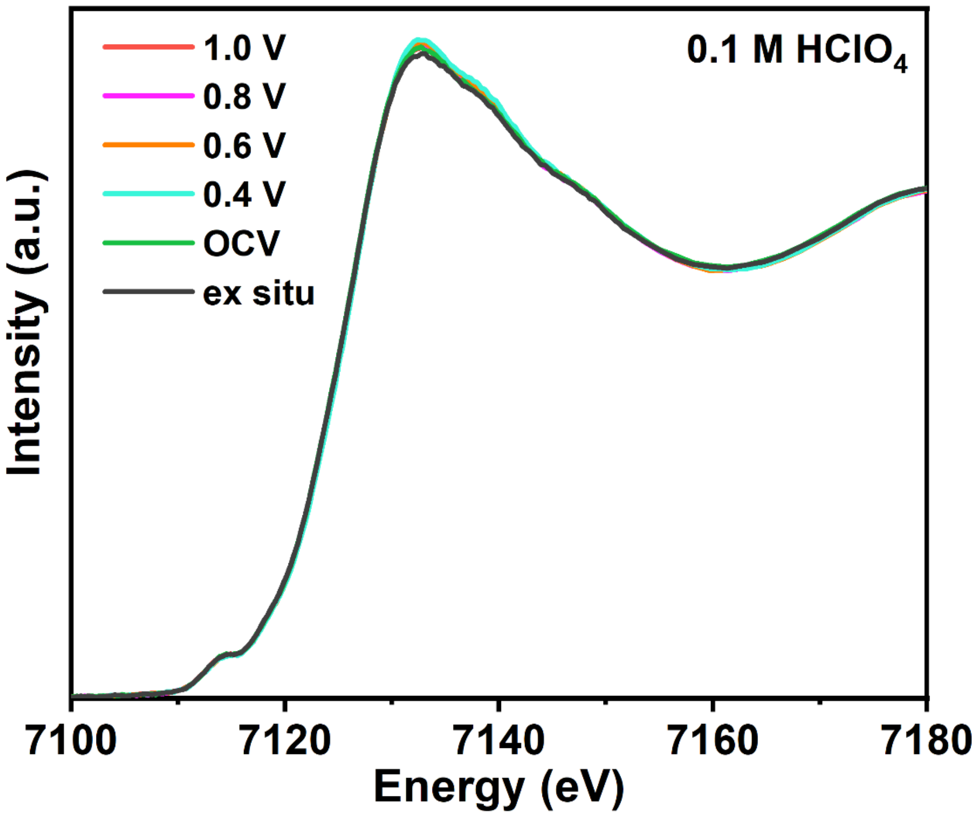

Supplementary Figure 38. *Operando* XANES of Fe K-edge for Fe SAs-HP in 0.1 M $HClO_4$. The XAS was conducted under O_2 saturated acidic electrolytes.

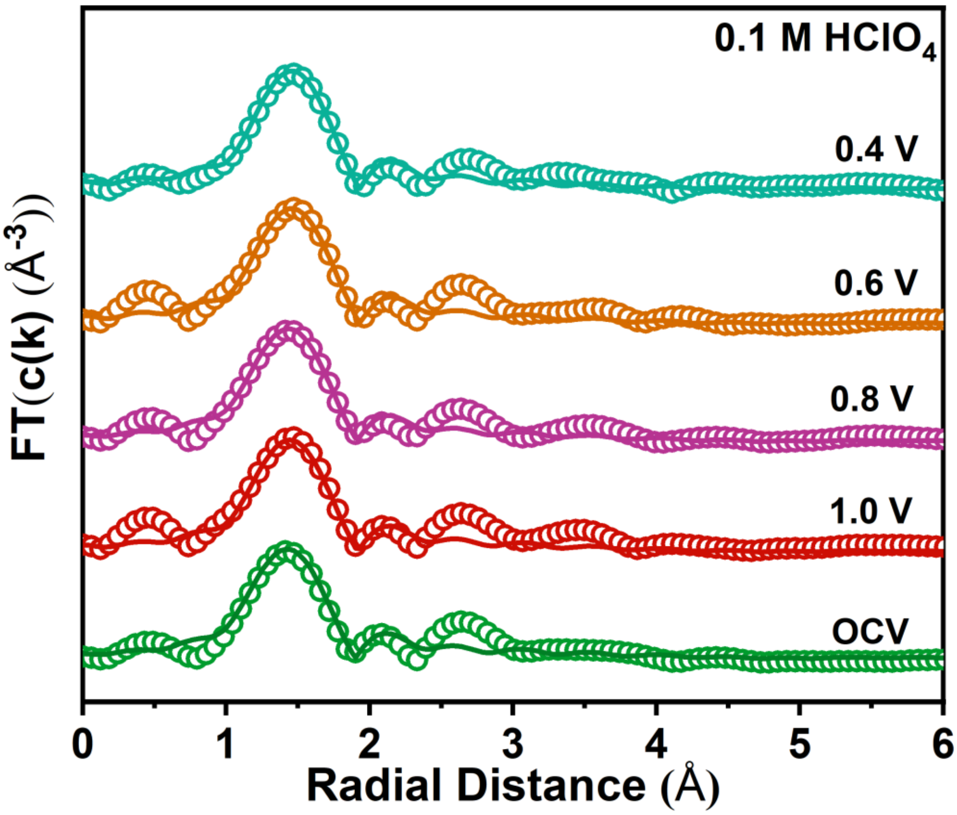

Supplementary Figure 39. corresponding FT-EXAFS fitting analysis of Fe SAs-HP under 0.1 M $HClO_4$.

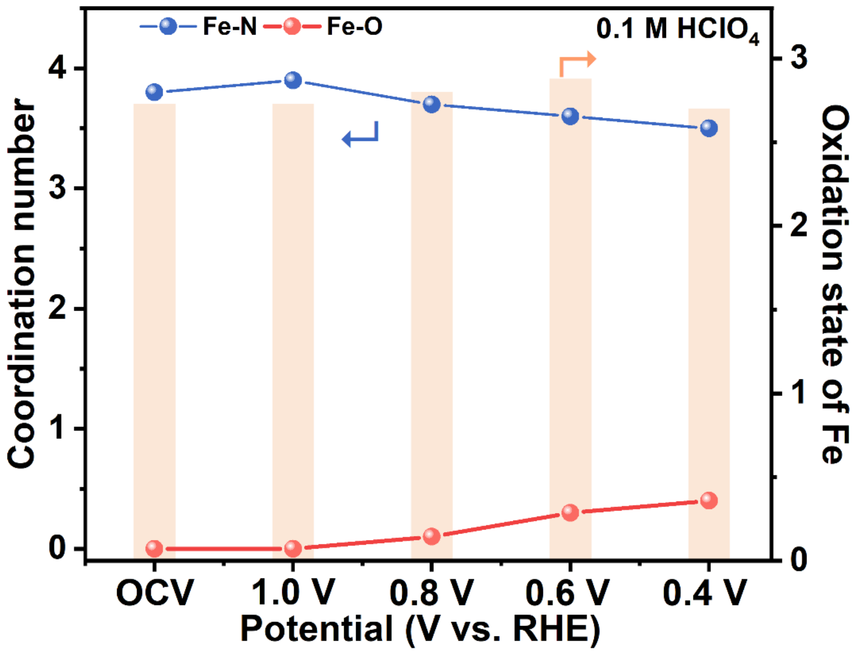

Supplementary Figure 40. Corresponding coordination number for Fe-N and Fe-O as well as oxidation state of Fe under acidic working conditions. Active Fe-N_4 centers remained the Fe-N_4 structure under acidic working conditions.

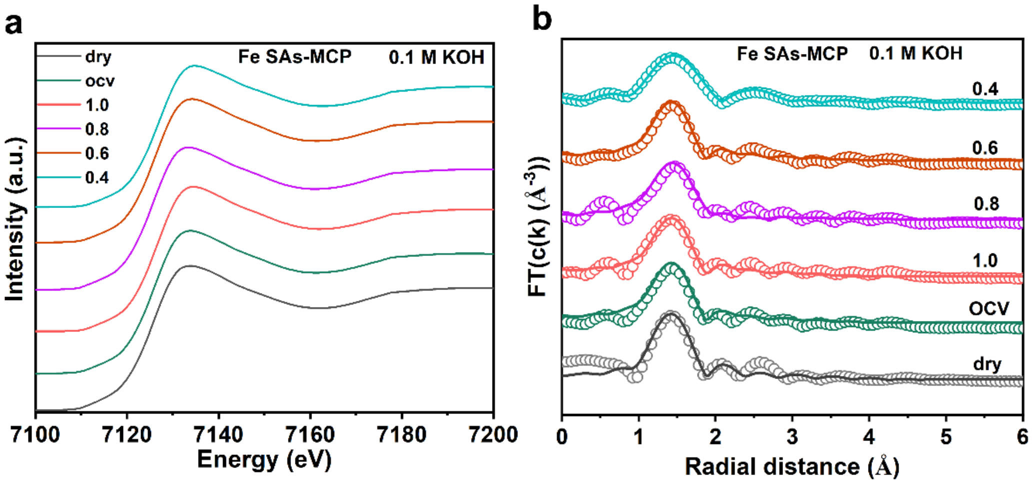

Supplementary Figure 41. *Operando* XAS characterizations for Fe SAs-MCP in 0.1 M KOH. (a) and (b) *Operando* XANES of Fe K-edge and corresponding FT-EXAFS fitting analysis for Fe SAs-MCP under 0.1 M KOH. The XAS was conducted under O_2 saturated alkaline electrolytes.

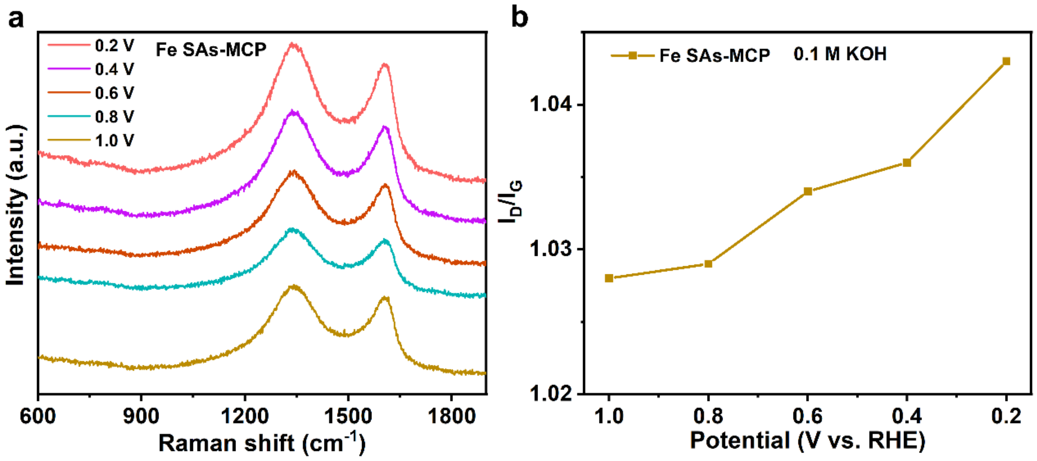

Supplementary Figure 42. *In situ* Raman for Fe SAs-MCP. (a) and (b) *In situ* Raman spectra of Fe SAs-MCP tested in 0.1 M KOH and corresponding I_D/I_G values with applied overpotentials. The I_D/I_G values increased with the applied overpotentials.

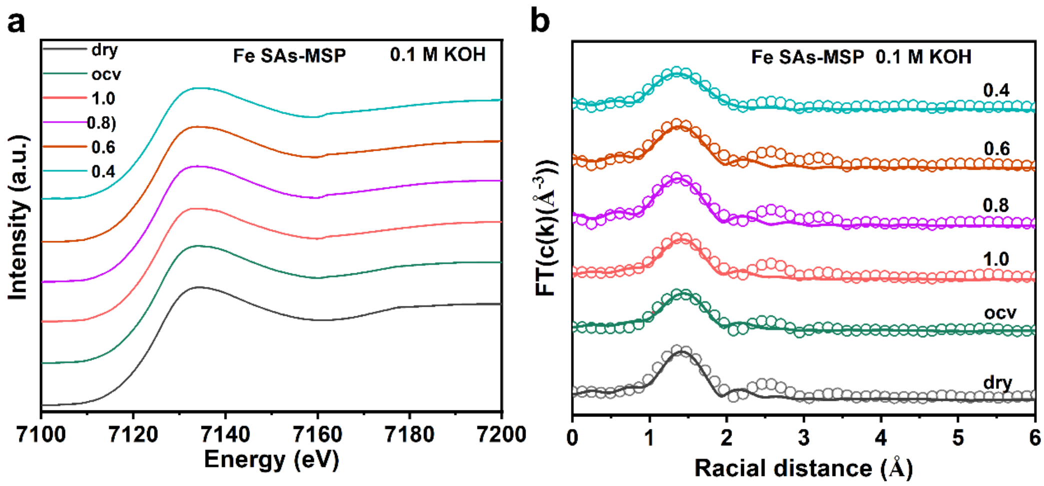

Supplementary Figure 43. *Operando* XAS characterizations for Fe SAs-MSP in 0.1 M KOH. (a) and (b) *Operando* XANES of Fe K-edge and corresponding FT-EXAFS fitting analysis for Fe SAs-MSP under 0.1 M KOH.

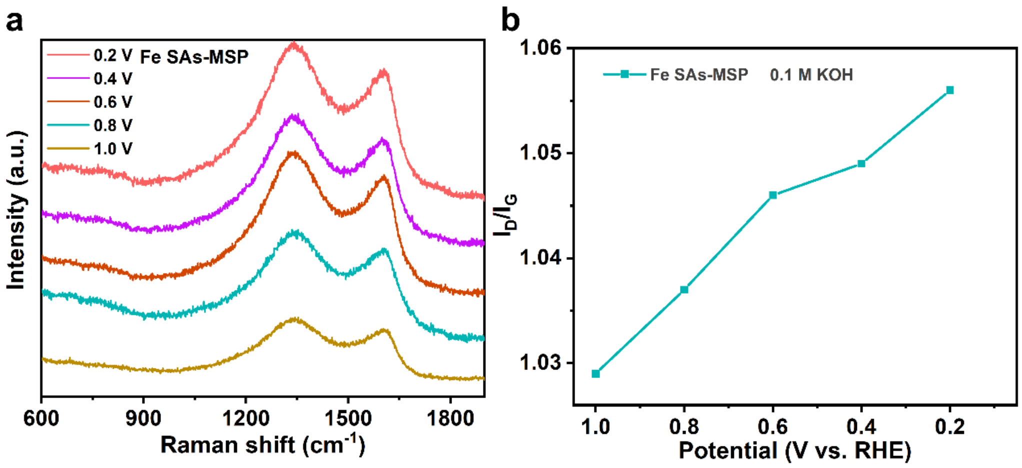

Supplementary Figure 44. *In situ* Raman for Fe SAs-MCP. (a) and (b) *In situ* Raman spectra of Fe SAs-MSP tested in 0.1 M KOH and corresponding I_D/I_G values with applied overpotentials.

Supplementary Tables

Supplementary Table 1. BET surface area (S_{BET}) and micropore area (S_{mcp}) of as-prepared catalysts.

Catalysts	S_{BET}	S_{mic}	**S_{msp}**	**S_{mic}/S_{BET}**
Fe SAs-HP	578.9	344.5	**234.4**	**59.5%**
Fe SAs-MCP	312.7	270.1	**42.6**	**86.4%**
Fe SAs-MSP	137.9	54.1	**83.8**	**39.2%**

Considering the relative low proportions of slit-type pores of Fe SAs-MCP and Fe SAs-MSP and the key role of microporous sites in regulating the catalytic performance, the proportion of S_{mcp} (S_{mcp}/S_{BET}) was applied as an indicator to qualify the effect of pore size.

Supplementary Table 2. Fe contents of synthesized catalysts determined by ICP-MS (weight ratio, wt%)

Catalysts	Fe/ wt%
Fe SAs-HP	0.7231
Fe SAs-MCP	0.4299
Fe SAs-MSP	0.2046
Fe SAs-in plane	0.0417

Supplementary Table 3. The Fe contents determined by ICP-MS of electrolytes after harsh stability tests for as-prepared catalysts.

Samples	Fe contents in electrolytes (mg/L)
Fresh electrolytes	0.0651
Fe SAs-HP	0.1628
Fe SAs-MCP	0.1793
Fe SAs-MSP	0.2673

Fe SAs-MSP had the highest Fe content after harsh stability tests, indicating the disengage of Fe atoms from mesoporous sites.

Supplementary Table 4. Alkaline ORR performance and peak power density of as-assembled zinc-air batteries (ZAB) of recent reported Catalysts.

Catalyst	E_{onset} (V vs. RHE)	$E_{1/2}$ (V vs. RHE)	Peak power density of ZABs (mW cm^{-2})	Catalyst loadings (mg cm^{-2}, RDE)	Reference
Fe SAs-HP	1.06	0.94	254.2	1.0	This work
Fe-N/P-C-700	0.94	0.87	133.2	0.6	*J. Am. Chem. Soc.* **142,** 2404−2412 (2020)[1]
Fe_H-N-C	-	0.91	225	0.6	*Adv. Mater.* **35,** 2210714 (2023)[2]
FeN_3OS	1.01	0.874	-	0.4	*Angew. Chem. Int. Ed.* **60,** 25296 –25301 (2021)[3]
Fe_1Se_1-NC	1.0	0.88	-	0.2	*Appl. Catal. B-Environ*, **308,** 121206 (2022)[4]
Fe/Meso-NC-1000	0.97	0.885	188.4	0.3	*Adv. Mater.* **34,** 2107291 (2022)[5]
Fe-N-GDY	1.05	0.89	249	0.4	*Angew. Chem. Int. Ed.* **61**, e202208238 (2022)[6]
OAC	0.98	0.85	113	0.3	*Appl. Catal. B-Environ*. 305, 121058 (2022)[7]
Fe-SA-NSFC	1.01	0.91	247.7	0.5	*Nat. Commun.* **11,** 5892 (2020)[8]
Fe SAs-Fe_2P NPs/NPCFs-2.5	1.03	0.91	236	0.5	*Adv. Mater.* **34,** 2203621 (2022)[9]
Co_2/Fe-N@CHC	1.03	0.915	232.4	0.3	*Adv. Mater.* **33,** 2104718 (2021)[10]
Fe,Mn/N-C	0.979	0.928	160.8	0.1	*Nat. Commun.* **12,** 1734 (2021)[11]

Fe,P-DAS@MPC	1.02	0.92	230	0.255	*Adv. Energy Mater.* **13**, 2203611 (2022)[12]

Supplementary Table 5. Acidic ORR performance of recently reported Catalysts.

Catalyst	E_{onset} (V vs. RHE）	$E_{1/2}$ （V vs. RHE）	Catalyst loadings (mg cm^{-2}, RDE)	Reference
Fe SAs-HP	0.90	0.78	1.0	This work
Fe_1Se_1-NC	0.88	0.74	0.2	*Appl. Catal. B: Environ*. **308**, 121206 (2022)[4]
OAC	0.86	0.71	0.3	*Appl. Catal. B: Environ*. **305**, 121058 (2022)[7]
Fe/OES	0.80	0.71	0.4	*Angewandte Chemie*. **132**, 7454-7459 (2020)[13]
CoFe@C	0.80	0.70	0.408	*Angewandte Chemie*. **131**, 1997-2001 (2019)[14]
Fe/N-CNRs	0.89	0.73	0.4	*Adv. Funct. Mater*. **31**, 2008085 (2021)[15]
Fe/Ni-N-PCS DM-SAC	0.87	0.71	0.255	*J. Colloid Interf. Sci*. **633**, 828-835 (2023)[16]
Fe_{50}-N-C-900	0.88	0.73	0.1	*Small*. **14**, 1703118 (2018)[17]
Fe-N-C/N OMC	0.91	0.73	0.3	*Appl. Catal. B: Environ*. **280**, 119411 (2021)[18]
FeCu-DA/NC	0.89	0.78	0.5	*J. Mater. Chem. A*. **8**, 16994-17001 (2020)[19]

Supplementary Table 6. Comparison of TOF and mass activity at 0.8 V for Fe SAs-HP and reported catalysts.

catalysts	TOF (s^{-1})	Mass activity (A g_{Fe}^{-1})	reference
Fe SAs-HP	5.99	4.14 * 10^4	This work
Fe SACs	4.3	1.5 x 10^3 (GDE)	*Nat. Catal.* **4,** 615-622 (2021)[20]
Cyan-Fe-N-C	0.79	1.142*10^3	*Adv. Mater.* **35,** 2305945 (2023)[21]
TAP 900@Fe	0.087	4.0	*Adv. Mater.* **35,** 2211022 (2023)[22]
sur-FeN_4-HPC	1.01	16.5	*Energy Environ. Sci.* **15,** 2619 (2022)[23]
TPI@Z8(SiO_2)-650-C	1.63	-	*Nat. Catal.* **2,** 259-268 (2019)[24]
Fe-SA-NSFC	0.22	-	*Nat Commun.* **11,** 5892 (2020)[8]
LTHT-FeP aerogel	0.25	-	*Angew. Chem. Int. Ed.* **59,** 2483-2489 (2020)[25]

Supplementary Table 7. ***Operando*** **XAS analysis parameters of coordination number (CN) for active Fe sites for Fe SAs-HP in 0.1 M KOH under working conditions.**

Potentials	path	CN	R (Å)	dE	dW
OCV	Fe-N	3.9 (2)	1.94 (3)	-13.9 (5)	0.0091 (6)
1.0 V	Fe-N	3.8 (3)	1.94 (2)	-13.8 (5)	0.0091 (6)
	Fe-O	-	-	-	-
0.8 V	Fe-N	3.6 (2)	1.95 (5)	-14.3 (7)	0.0082 (8)
	Fe-O	0.3 (4)	2.03 (4)	-3.8 (6)	0.0088 (6)
0.6 V	Fe-N	3.1 (1)	1.97 (3)	-14.7 (7)	0.0091 (9)
	Fe-O	0.6 (3)	2.04 (3)	-4.2 (4)	0.0075 (8)
0.4 V	Fe-N	2.9 (3)	1.99 (4)	-15.7 (5)	0.0094 (7)
	Fe-O	0.8 (4)	2.03 (5)	-3.5 (8)	0.0079 (5)

The number in brackets represents deviation of obtained results. For example, 3.9 (2) means the CN is 3.9 ± 0.2 and 1.94 (3) means the R distance is 1.94 ± 0.03.

Supplementary Table 8. ***Operando*** **XAS analysis parameters of coordination number (CN) for active Fe sites for Fe SAs-HP in 0.1 M $HClO_4$ under working conditions.**

Potentials	path	CN	R (Å)	dE	dW
OCV	Fe-N	3.8 (4)	1.94 (3)	-15.9 (8)	0.0082 (6)
1.0 V	Fe-N	3.9 (4)	1.95 (2)	-14.2 (7)	0.0083 (4)
	Fe-O	-	-	-	-
0.8 V	Fe-N	3.7 (5)	1.95 (6)	-14.8 (7)	0.0087 (8)
	Fe-O	0.1 (3)	2.04 (4)	-1.7 (4)	0.0088 (6)
0.6 V	Fe-N	3.6 (6)	1.96 (5)	-13.1 (8)	0.0084 (9)
	Fe-O	0.3 (3)	2.04 (1)	-3.3 (6)	0.0075 (5)
0.4 V	Fe-N	3.5 (5)	1.97 (4)	-13.2 (7)	0.0081 (8)
	Fe-O	0.4 (5)	2.03 (4)	-0.5 (6)	0.0079 (5)

The number in brackets represents deviation of obtained results.

Supplementary Table 9. *Operando* XAS analysis parameters of coordination number (CN) for active Fe sites of Fe SAs-MCP in 0.1 M KOH under working conditions.

Potentials	path	CN	R (Å)	dE	dW
dry	Fe-N	3.8(2)	1.94(3)	-7.5(3)	0.0084(7)
ocv	Fe-N	3.8(2)	1.95(2)	-8.3(3)	0.0083(7)
1.0	Fe-N	3.7(4)	1.94(3)	-7.9(4)	0.0084(6)
	Fe-O	-	-	-	-
0.8	Fe-N	3.7(4)	1.95(5)	-8.6(5)	0.0081(5)
	Fe-O	0.3(4)	2.02(4)	-5.2(7)	0.0097(7)
0.6	Fe-N	3.2(4)	1.96(5)	-11.4(5)	0.0092(7)
	Fe-O	0.5(3)	2.03(3)	-5.7(5)	0.0095(6)
0.4	Fe-N	1.8(2)	1.94(4)	-10.7(6)	0.0089(7)
	Fe-O[1]	0.3(4)	2.03(3)	-4.5(5)	0.0079(7)
	Fe-O[2]	3.6(2)	2.13(3)	6.2(5)	0.0062(8)
	Fe-Fe	6.1(2)	3.08(4)	-1.8(7)	0.0095(7)

Fe-O[1]: Fe-O scattering path between active Fe centers and oxygenated intermediates.
Fe-O[2]:Fe-O scattering path between active Fe centers and lattice oxygen in FeO.

Supplementary Table 10. ***Operando*** **XAS analysis parameters of coordination number (CN) for active Fe sites of Fe SAs-MSP in 0.1 M KOH under working conditions.**

Potentials	path	CN	R (Å)	dE	dW
dry	Fe-N	3.5(4)	1.96(2)	-12.3(4)	0.0105(5)
ocv	Fe-N	3.5(3)	1.95(2)	-10.2(5)	0.0106(4)
1.0	Fe-N	3.5(4)	1.96(4)	-11.5(5)	0.0106(7)
	Fe-O	-	-	-	-
0.8	Fe-N	3.4(4)	1.96(5)	-12.5(5)	0.0111(5)
	Fe-O	0.3(3)	2.02(4)	-5.0(6)	0.0083(5)
0.6	Fe-N	3.1(2)	1.96(3)	-13.4(4)	0.0096(5)
	Fe-O	0.5(2)	2.02(2)	-5.1(5)	0.0081(6)
0.4	Fe-N	1.7(3)	1.92(2)	-11.7(6)	0.0089(6)
	Fe-O	0.5(2)	2.01(4)	-6.5(5)	0.0086(6)

Supplementary References

1. Yuan, K. et al. Boosting oxygen reduction of single iron active sites via geometric and electronic engineering: nitrogen and phosphorus dual coordination. *J. Am. Chem. Soc.* **142**, 2404-2412 (2020).
2. Tian, H. et al. High durability of Fe-N-C single-atom catalysts with carbon vacancies toward the oxygen reduction reaction in alkaline media. *Adv. Mater.* **35**, 2210714 (2023).
3. Yu, L., Li, Y. & Ruan, Y. Dynamic control of sacrificial bond transformation in the Fe-N-C single-atom catalyst for molecular oxygen reduction. *Angewandte Chemie*. **133**, 25500-25505 (2021).
4. Chen, Z. et al. Boosting oxygen reduction reaction with Fe and Se dual-atom sites supported by nitrogen-doped porous carbon. *Applied Catalysis B: Environmental*. **308**, 121206 (2022).
5. Zhao, S. N., Li, J. K., Wang, R., Cai, J. & Zang, S. Q. Electronically and geometrically modified single-atom Fe sites by adjacent Fe nanoparticles for enhanced oxygen reduction. *Adv. Mater.* **34**, 2107291 (2022).
6. Li, M., Lv, Q., Si, W., Hou, Z. & Huang, C. Sp-hybridized nitrogen as new anchoring sites of iron single atoms to boost the oxygen reduction reaction. *Angewandte Chemie*. **61**, e202208238 (2022).
7. Deng, L. et al. Restricted diffusion preparation of fully-exposed Fe single-atom catalyst on carbon nanospheres for efficient oxygen reduction reaction. *Applied Catalysis B: Environmental*. **305**, 121058 (2022).
8. Zhou, Y. et al. Multilayer stabilization for fabricating high-loading single-atom catalysts. *Nat. Commun.* **11**, 5892 (2020).
9. Pan, Y. et al. Construction of N, P co-doped carbon frames anchored with Fe single atoms and Fe_2 P nanoparticles as a robust coupling catalyst for electrocatalytic oxygen reduction. *Adv. Mater.* **34**, 2203621 (2022).
10. Wang, Z. et al. Atomically dispersed Co_2-N_6 and Fe-N_4 costructures boost oxygen reduction reaction in both alkaline and acidic media. *Adv. Mater.* **33**, 2104718 (2021).
11. Yang, G. et al. Regulating Fe-spin state by atomically dispersed Mn-N in Fe-N-C catalysts with high oxygen reduction activity. *Nat. Commun.* **12**, 1734 (2021).
12. Zong, L. et al. Promoting oxygen reduction reaction on atomically dispersed Fe sites via

establishing hydrogen bonding with the neighboring P atoms. *Adv. Energy Mater.* **13**, 2203611 (2023).

13. Hou, C. C. et al. Single-atom iron catalysts on overhang-eave carbon cages for high-performance oxygen reduction reaction. *Angewandte Chemie*. **132**, 7454-7459 (2020).
14. Zhao, R. et al. Puffing up energetic metal-organic frameworks to large carbon networks with hierarchical porosity and atomically dispersed metal sites. *Angewandte Chemie*. **131**, 1997-2001 (2019).
15. Gong, X. et al. Self-templated hierarchically porous carbon nanorods embedded with atomic Fe-N_4 active sites as efficient oxygen reduction electrocatalysts in Zn-air batteries. *Adv. Funct. Mater.* **31**, 2008085 (2021).
16. Zhao, J. et al. Synthesis of dual-metal single atom in porous carbon with efficient oxygen reduction reaction in both acidic and alkaline electrolytes. *J. Colloid Interf. Sci.* **633**, 828-835 (2023).
17. Fu, S. et al. Porous carbon-hosted atomically dispersed iron-nitrogen moiety as enhanced electrocatalysts for oxygen reduction reaction in a wide range of pH. *Small*. **14**, 1703118 (2018).
18. Han, J. et al. 3D N-doped ordered mesoporous carbon supported single-atom Fe-N-C catalysts with superior performance for oxygen reduction reaction and zinc-air battery. *Applied Catalysis B: Environmental*. **280**, 119411 (2021).
19. Du, C. et al. A Cu and Fe dual-atom nanozyme mimicking cytochrome c oxidase to boost the oxygen reduction reaction. *J. Mater. Chem. A*. **8**, 16994-17001 (2020).
20. Jin, Z. et al. Understanding the inter-site distance effect in single-atom catalysts for oxygen electroreduction. *Nat. Catal*. **4**, 615-622 (2021).
21. Yuan, L. J. et al. d-orbital electron delocalization realized by axial Fe_4C atomic clusters delivers high-performance Fe-N-C catalysts for oxygen reduction reaction. *Adv. Mater.* **35**, (2023).
22. Barrio, J. et al. FeNC Oxygen Reduction Electrocatalyst with High Utilization Penta ‐ Coordinated Sites. *Adv. Mater.* **35**, 2305945 (2023).
23. Chen, G. et al. Highly accessible and dense surface single metal FeN_4 active sites for promoting the oxygen reduction reaction. *Energ. Environ. Sci.* **15**, 2619-2628 (2022).
24. Wan, X. et al. Fe-N-C electrocatalyst with dense active sites and efficient mass transport for high-performance proton exchange membrane fuel cells. *Nat. Catal*. **2**, 259-268 (2019).

25. Zion, N., Cullen, D. A., Zelenay, P. & Elbaz, L. Heat-treated aerogel as a catalyst for the oxygen reduction reaction. *Angewandte Chemie International Edition*. **59**, 2483-2489 (2020).
